# A pathogen effector co-opts a host RabGAP protein to remodel pathogen interface and subvert defense-related secretion

**DOI:** 10.1126/sciadv.ado9516

**Published:** 2024-10-04

**Authors:** Enoch Lok Him Yuen, Yasin Tumtas, Freddie King, Tarhan Ibrahim, Lok I Chan, Edouard Evangelisti, Frej Tulin, Jan Skłenar, Frank L. H. Menke, Sophien Kamoun, Doryen Bubeck, Sebastian Schornack, Tolga Osman Bozkurt

**Affiliations:** ^1^ Department of Life Sciences, Imperial College London, London SW7 2AZ, UK.; ^2^ Sainsbury Laboratory (SLCU), University of Cambridge, Cambridge CB2 1LR, UK.; ^3^ Université Côte d’Azur, INRAE UMR 1355, CNRS UMR 7254, Institut Sophia Agrobiotech (ISA), 06903 Sophia Antipolis, France.; ^4^ Department of Plant Biology, Carnegie Institution for Science, Stanford, CA 94305, USA.; ^5^ The Sainsbury Laboratory, University of East Anglia, Norwich Research Park, Norwich NR4 7UH, UK.

## Abstract

Pathogens have evolved sophisticated mechanisms to manipulate host cell membrane dynamics, a crucial adaptation to survive in hostile environments shaped by innate immune responses. Plant-derived membrane interfaces, engulfing invasive hyphal projections of fungal and oomycete pathogens, are prominent junctures dictating infection outcomes. Understanding how pathogens transform these host-pathogen interfaces to their advantage remains a key biological question. Here, we identified a conserved effector, secreted by plant pathogenic oomycetes, that co-opts a host Rab GTPase-activating protein (RabGAP), TOPGAP, to remodel the host-pathogen interface. The effector, PiE354, hijacks TOPGAP as a susceptibility factor to usurp its GAP activity on Rab8a, a key Rab GTPase crucial for defense-related secretion. By hijacking TOPGAP, PiE354 purges Rab8a from the plasma membrane, diverting Rab8a-mediated immune trafficking away from the pathogen interface. This mechanism signifies an uncanny evolutionary adaptation of a pathogen effector in co-opting a host regulatory component to subvert defense-related secretion, thereby providing unprecedented mechanistic insights into the reprogramming of host membrane dynamics by pathogens.

## Teaser

A pathogen hijacks a plant protein to avoid immune defenses by reprogramming host intracellular trafficking.

## INTRODUCTION

Plants are equipped with a dynamic innate immune system to sense and confront pathogens. This system fundamentally relies on endomembrane trafficking, which facilitates a hostile environment against pathogens by directing immune components, such as pathogenesis-related (PR) proteins, to the pathogen interface. Consistent with this notion, a growing number of studies have revealed pathogen manipulation of plant vesicle trafficking as a ubiquitous infection strategy (*1*–*6*).

Pathogens intimately interact with plant cells via specialized structures that facilitate the transfer of effector proteins and the uptake of nutrients. Filamentous plant pathogens, including oomycetes and fungi, project hyphal extensions that breach the cell wall and penetrate host cells. At these junctures, plants mount targeted immune responses, including cellular reinforcements, the secretion of defense molecules, and the relocation of organelles (*7*–*13*). Filamentous pathogens have developed strategies to overcome these defenses, forming specialized infection structures like haustoria or infection vesicles (formed by oomycete pathogens), which are accommodated inside host cells. These infection structures are enveloped by plant-derived membranes with unique biochemical compositions, often lacking transmembrane proteins including pattern recognition receptors (PRRs), delineating a polarized membrane interface (*14*, *15*). At these interfaces, pathogens are thought to manipulate the environment, creating safe niches for efficient effector delivery and nutrient absorption (*16*). However, the regulatory mechanisms governing the trafficking of immune components at the host-pathogen interface and the extent to which they are manipulated by pathogen effectors remain largely unknown.

Rab guanosine triphosphatases (GTPases) (Rabs) are integral to vesicle trafficking and immunity, mediating the movement and fusion of vesicles with membrane compartments (*17*–*19*). While the immune functions of plant Rabs remain largely unknown, members of the Rab8 and Rab11 have been identified to contribute to pathogen resistance by facilitating defense-related secretion (*3*, *4*, *20*). Rabs function as molecular switches cycling between guanosine 5′-triphosphate (GTP)–bound active and guanosine diphosphate (GDP)–bound inactive states. Their activation is regulated by guanine nucleotide exchange factors, which facilitate GTP loading, and their inactivation is mediated by GTPase-activating proteins (GAPs), which accelerate GTP hydrolysis (*21*). Most RabGAPs are characterized by the Tre2/Bub2/Cdc16 (TBC) domain featuring dual catalytic fingers accelerating the GTP hydrolysis of their cognate Rabs (*22*), thereby controlling their localization and functions. Although a few RabGAPs have been implicated in immunity (*23*, *24*), the mechanisms behind their action, their specific Rab substrates, and the trafficking pathways that they regulate in immune responses remain largely unexplored in both plants and animals.

The critical role of membrane trafficking in plant pathogen defense is increasingly evident, with diverse pathogens deploying effectors that virtually target every facet of vesicle trafficking (*5*, *6*, *10*, *16*, *25*–*27*). Proteomic screens have highlighted the strategic targeting of the host vesicle trafficking system by *Phytophthora* pathogen (*6*, *28*). Notably, these pathogens deploy effectors converging on key Rab GTPases, like Rab8 and Rab11, which are integral to defense-related secretion (*3*, *4*, *20*). However, the detailed mechanisms of these interactions and their impact on host membrane dynamics are still not fully understood. Despite extensive documentation of effectors targeting host Rab GTPases in plant and animal pathosystems, the potential targeting of RabGAPs by pathogen effectors remains an unexplored area. This is particularly intriguing given the crucial role of RabGAPs in regulating Rab functions.

Here, we elucidate an unprecedented mechanism used by a conserved effector family from the *Phytophthora* species, notably PiE354 and its homologs, in reconfiguring host cell membrane dynamics at the pathogen interface. PiE354 adeptly co-opts the host RabGAP protein TOPGAP to harness its GAP activity on Rab8a. This manipulation expels Rab8a from the plasma membrane, redirecting Rab8a-mediated secretion of antimicrobial compounds away from the site of pathogen attack. Our findings suggest a detailed mechanistic model where PiE354 physically perturbs the TOPGAP-Rab8a complex, leveraging the GAP activity of TOPGAP to subvert Rab8a-mediated immune trafficking. Our research uncovers a sophisticated strategy used by pathogen effectors, demonstrating how they exploit the catalytic functions of a host transport regulator to effectively remodel host membrane dynamics to subvert immune responses.

## RESULTS

### A plant RabGAP protein is targeted by a conserved *Phytophthora* effector

To elucidate how *Phytophthora* species manipulate vesicle trafficking pathways, we focused on identifying effectors that target the plant endomembrane transport machinery. As part of an unbiased yeast two-hybrid (Y2H) screen, we discovered that the *Phytophthora palmivora* effector TIKI (trafficking interference and tissue-killing effector; PLTG_0964243, table S1) associates with a *Nicotiana benthamiana* TBC-containing RabGAP protein, termed TOPGAP (target of *Phytophthora* GAP, Nbe.v1.s00100g29830; table S2). TIKI belongs to the RXLR family of effectors, which are modular proteins characterized by a secretion signal peptide, an RXLR motif, and a functional effector domain (*29*). TIKI homologs are found exclusively within the *Phytophthora* genus, including the pathogens *Phytophthora nicotianae*, *Phytophthora lilii*, *Phytophthora infestans*, *P. palmivora*, and *Phytophthora cactorum* (fig. S1, A and B), with amino acid sequence similarities in the effector domains ranging from 50.0 to 62.8%. To corroborate the Y2H results, we conducted an immunoprecipitation-mass spectrometry (IP-MS) analysis, which again pinpointed TOPGAP as a candidate interactor of TIKI (table S3).

**Fig. 1.**
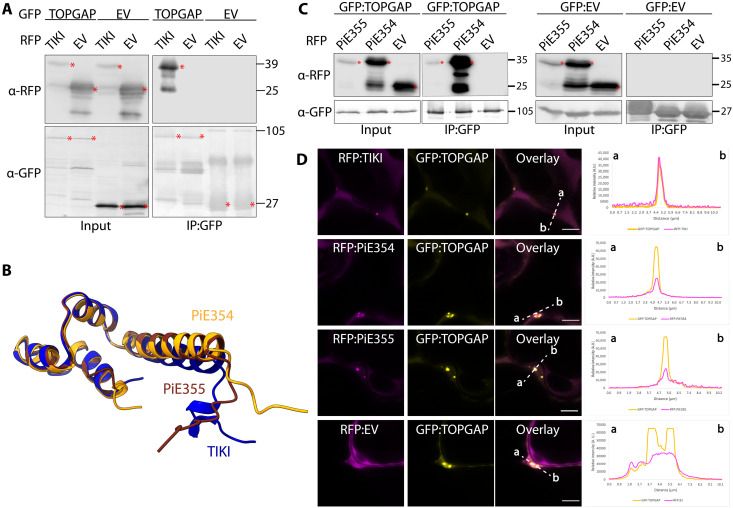
Conserved effectors from *Phytophthora* species target TOPGAP. (**A**) TIKI interacts with TOPGAP *in planta*. RFP:TIKI or RFP:EV was transiently co-expressed with GFP:TOPGAP or GFP:EV. IPs were obtained with anti-GFP antibody. Total protein extracts were immunoblotted. Red asterisks indicate expected band sizes. Numbers on the right indicate kilodalton values. (**B**) Structural alignment of the effectors TIKI (blue) from *P. palmivora* and PiE354 (orange) and PiE355 (brown) from *P. infestans*. Structural predictions were obtained via AF2. The model shows overall structural conservation of the effectors, without their secretion signals and RXLR motifs, as these regions are cleaved off and are not part of the mature effector protein and are not required for their virulence function (*53*). (**C**) PiE355 and PiE354 interact with TOPGAP in planta. GFP:TOPGAP was transiently co-expressed with RFP:PiE355, RFP:PiE354, or RFP:EV. IPs were obtained with anti-GFP antibody. Total protein extracts were immunoblotted. Red asterisks indicate expected band sizes. Numbers on the right indicate kilodalton values. (**D**) TOPGAP colocalizes with TIKI, PiE354, and PiE355 in puncta in planta. Confocal micrographs of *N. benthamiana* leaf epidermal cells transiently expressing either RFP:TIKI, RFP:PiE354, RFP:PiE355, or RFP:EV, with GFP:TOPGAP. Presented images are single-plane images. Overlay panel transects correspond to line intensity plots showing relative fluorescence across the marked distance. Scale bars, 5 μm. A.U., arbitrary units.

We validated the association between TIKI and TOPGAP through in planta reverse co-immunoprecipitation (co-IP), pulling down N-terminally green fluorescent protein (GFP)–tagged TOPGAP with a plant expression construct of TIKI effector domain N-terminally tagged with red fluorescent protein (RFP) (Fig. 1A). In *N. benthamiana* expression assays, we observed that TIKI triggers plant cell death (fig. S2A), presenting challenges for conducting accurate biochemical and cellular biology assays. However, the two TIKI homologs from *P. infestans* (fig. S2B), named PiE354 (*P. infestans* effector 354, PITG_04354; table S1) and PiE355 (*P. infestans* effector 355, PITG_04355; table S1), showed varying cell death responses. PiE355 induced less severe cell death than TIKI, whereas PiE354 showed no visible symptoms (fig. S2C), presenting an excellent opportunity for elucidating the functions of these effectors. AlphaFold2 (AF2) structural predictions showed notable similarity among these three *Phytophthora* effectors, indicated by a low root mean square deviation value of 0.810 and 0.374 when comparing TIKI to PiE354 and PiE355, respectively, hinting at a conserved mode of action (Fig. 1B and fig. S2D). Consistent with this notion, co-IP experiments using protein extracts from *N. benthamiana* demonstrate that GFP:TOPGAP interacts with both RFP:PiE354 and RFP:PiE355 but not with RFP:empty vector (EV) control (Fig. 1C). Conversely, the GFP:EV control did not interact with any of the effectors (Fig. 1C). Consistent with these findings, confocal microscopy analysis revealed that TOPGAP colocalizes with all three effectors (TIKI, PiE354, and PiE355) at discrete punctate structures and in the cytosol (Fig. 1D). It is worth noting that, while PiE354 exhibited puncta formation, the frequency was less than the other two effectors (fig. S2E). In addition, PiE354 exhibited a similar localization pattern to TOPGAP in infected cells, remaining primarily cytoplasmic and occasionally forming puncta around pathogen haustoria (fig. S2F). Together, these results show that TIKI and its *P. infestans* homologs, PiE354 and PiE355, target TOPGAP in host plants.

### PiE354 targets the N-terminal RBD of TOPGAP

**Fig. 2.**
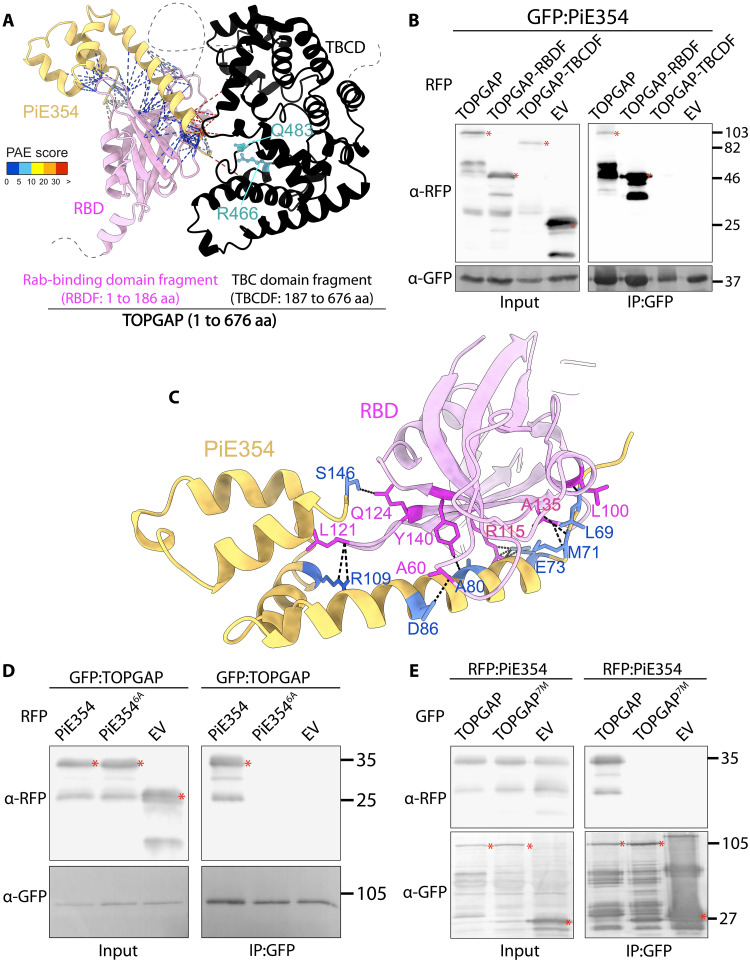
PiE354 targets the N-terminal RBD fragment of TOPGAP. (**A**) AF2-M–predicted model of PiE354 targeting TOPGAP. PiE354, RBD of TOPGAP, and TBCD of TOPGAP are depicted in yellow, pink, and black, respectively. The key residues responsible for the GAP activity of TOPGAP, R466 and Q483, are highlighted in cyan. The putative bonds between PiE354 and TOPGAP are predicted by ChimeraX with a distance of 5 Å. The colors of the bonds are based on the AF2-calculated predicted aligned error (PAE) score. A low PAE score, indicating high-confidence contacts, is shown by blue lines. A high PAE score, indicating low-confidence contacts, is shown by red lines. The curved gray dashed lines indicate the predicted disordered regions in TOPGAP. aa, amino acids. (**B**) PiE354 interacts with full-length TOPGAP and RBDF of TOPGAP but not with TBCDF of TOPGAP and EV. GFP:PiE354 was transiently co-expressed with RFP:TOPGAP, RFP:RBDF, RFP:TBCDF, or RFP:EV. (**C**) AF2-M–predicted model of PiE354 (yellow) targeting the RBD of TOPGAP (pink), depicting their interacting residues. The bonds between PiE354 and TOPGAP are predicted by PyMOL with a distance of 3 Å. The PiE354-RBD interaction interface consists of seven key residues on both proteins, colored as blue on PiE354 and as magenta on RBD. (**D**) PiE354 targets TOPGAP through six key residues on PiE354. GFP:TOPGAP was transiently co-expressed with either RFP:PiE354, RFP:PiE354^6A^, or RFP:EV. (**E**) TOPGAP interacts with PiE354 through seven key residues on TOPGAP. RFP:PiE354 was transiently co-expressed with either GFP:TOPGAP, GFP:TOPGAP^7M^, or GFP:EV. For all co-IP assays, IPs were obtained with anti-GFP antibody. Total protein extracts were immunoblotted. Red asterisks indicate expected band sizes. Numbers on the right indicate kilodalton values.

We next investigated the mechanisms by which PiE354 interacts with TOPGAP. We chose PiE354 because, unlike its homologs TIKI and PiE355, it does not induce cell death in plants, thus avoiding complications in physiological and functional analyses (fig. S2C). Taking advantage of AF2, we first visualized the protein architecture of TOPGAP. This analysis revealed a domain of unknown function, DUF3548, at the N terminus, and a TBC domain (TBCD) near the C terminus (fig. S3A). The DUF3548, although not fully characterized, has been reported to function as a Rab-binding domain (RBD) in the human TBC-RabGAP protein RUTBC2 (*30*). Notably, AF2-multimer (AF2-M) structural predictions of the TOPGAP-PiE354 complex indicated that PiE354 establishes multiple high-confidence contacts with the candidate RBD (DUF3548) and a few low confidence contacts with the TBCD, spanning a distance of about 5 Å (Fig. 2A and fig. S3B), suggesting that the effector targets the RBD of TOPGAP. This finding aligns with our Y2H results (table S2), which indicated that the N-terminal region of TOPGAP is sufficient for binding TIKI.

To experimentally validate the predicted binding interface between PiE354 and TOPGAP, we designed two plant expression constructs encoding N- and C-terminal fragments of TOPGAP. The first construct included the N-terminal RBD fragment, denoted RBDF (1 to 186), and the second comprised the C-terminal TBCD fragment, named TBCDF (186 to 676). The TBCDF construct triggered a slight cell death response, typically noticeable within 3 to 4 days of transient expression (fig. S3C). Nevertheless, Western blot analysis confirmed the successful in planta expression of both TOPGAP fragments, although the protein levels of TBCDF were slightly lower compared to those of RBDF and full-length TOPGAP (Fig. 2B). Our pull-down assays, conducted with protein extracts from *N. benthamiana*, revealed a strong interaction between PiE354 and the RBDF construct. In contrast, we did not detect any interaction between PiE354 and the TBCDF construct. Notably, this finding is in line with our Y2H results (table S2) and AF2-M predictions (Fig. 2A), confirming that PiE354 specifically targets the N-terminal RBD fragment of TOPGAP.

The AF2-M analysis with stringent parameters indicated seven crucial residues on PiE354 (L69, M71, E73, A80, D86, R109, and S146) that might be pivotal for its interaction with TOPGAP (Fig. 2C and fig. S3D). In addition, AF2-M predicts similar binding interfaces among TOPGAP-PiE354, TOPGAP-PiE355, and TOPGAP-TIKI, with slight variations in the number of contacts made (fig. S3, E and F). To characterize the interaction between PiE354 and TOPGAP further, we mutated the predicted key binding residues on PiE354. Because one of these residues was already encoding alanine (A15), we created a 6A mutant of PiE354 (PiE354^6A^) by substituting the other six residues for alanine. Our co-IP assays confirmed that TOPGAP interacts with PiE354, but not with the PiE354^6A^ mutant or EV (Fig. 2D). This result underscores the critical role of these six residues in PiE354 for its interaction with TOPGAP. Our confocal microscopy analyses further support this notion, showing colocalization of TOPGAP with PiE354 in puncta, whereas no puncta colocalization was evident with the PiE354^6A^ mutant, mirroring the behavior of the EV control (fig. S4A).

To further elucidate the effector targeting mechanism, we conducted reciprocal mutation experiments focusing on the AF2-predicted binding interface on the host target, TOPGAP. The AF2-M analysis indicated seven key residues on TOPGAP (L100, A135, R115, Y140, A60, L121, and Q124) important for binding with PiE354 (Fig. 2C and fig. S3D). Because A60 and A135 were already encoding alanine, we engineered a mutant, named TOPGAP^7M^, featuring substitutions of non-alanine residues to alanine and alanine residues to glycine, resulting in a total of seven mutations. We confirmed the expression of GFP:TOPGAP^7M^ using confocal microscopy, displaying similar characteristics to the wild-type (WT) GFP:TOPGAP protein, including cytoplasmic localization and puncta formation (fig. S4B). Our subsequent co-IP assays demonstrated that the TOPGAP^7M^ mutant was unable to interact with PiE354 (Fig. 2E), highlighting the critical role of these seven residues in TOPGAP for PiE354 targeting. Confocal microscopy analysis provide further support for this notion, as TOPGAP^7M^ did not colocalize with any of the effectors PiE354, PiE355, and TIKI in punctate structures (fig. S4C). This analysis provides crucial insights into the binding mechanism between PiE354 and its host target TOPGAP, identifying key residues in both proteins that are collectively required for their interaction.

### TOPGAP negatively regulates plant immunity and immune-related secretion in a GAP-dependent manner

**Fig. 3.**
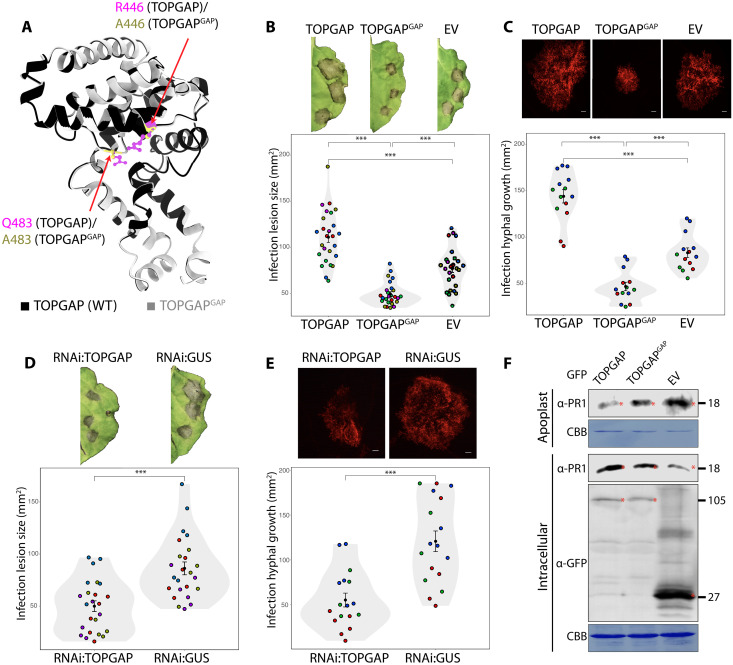
TOPGAP negatively regulates plant immunity through its GAP function. (**A**) Structural alignment of TOPGAP and its GAP mutant TOPGAP^GAP^ using AF2 predictions. (**B**) *N. benthamiana* leaves expressing TOPGAP, TOPGAP^GAP^, or EV control were infected with *P. infestans*, and pathogen growth was calculated by measuring infection lesion size at 8 days postinoculation (dpi). (**C**) *N. benthamiana* leaves expressing TOPGAP, TOPGAP^GAP^, or EV control were infected with tdTomato-expressing *P. infestans*, and pathogen growth was calculated by measuring hyphal growth using fluorescence stereomicroscope at 5 dpi. (**D**) *N. benthamiana* leaves expressing RNAi:TOPGAP or RNAi:GUS control were infected with *P. infestans*, and pathogen growth was calculated by measuring infection lesion size at 8 dpi. (**E**) *N. benthamiana* leaves expressing RNAi:TOPGAP or RNAi:GUS control were infected with tdTomato-expressing *P. infestans*, and pathogen growth was calculated by measuring hyphal growth using fluorescence stereomicroscope at 5 dpi. [(B) to (E)] Each color represents an independent biological replicate. Each dot represents the average of three infection spots on the same leaf. Statistical differences were analyzed by Student’s *t* test or Mann-Whitney *U* test in R. Scale bars, 10 μm. Measurements were highly significant when ***P < 0.001. Detailed statistical analyses can be found in table S9. (**F**) *N. benthamiana* leaves were agroinfiltrated to express GFP:TOPGAP, GFP:TOPGAP^GAP^, or GFP:EV. The infiltrated leaves were challenged with *P. infestans* extract at 3 dpi, and proteins were extracted from the apoplast and leaf tissue at 4 dpi and immunoblotted. Red asterisks show expected band sizes. Numbers on the right indicate kilodalton values. CBB, Coomassie Brilliant Blue staining.

Convergence of a conserved *Phytophthora* effector on TOPGAP hints at a key regulatory role of this RabGAP in immune-related subcellular trafficking. To assess the impact of TOPGAP on plant immunity, we conducted infection assays with *P. infestans* upon overexpression or silencing of TOPGAP in *N. benthamiana*. The dual catalytic fingers of TOPGAP, crucial for stimulating GTP hydrolysis of Rab GTPases, are located at R446 and Q483 positions within the TBCD (Fig. 2A). We created the TOPGAP GAP mutant (TOPGAP^GAP^) by the dual mutations at R446A and Q483A positions (Fig. 3A and fig. S5A), which typically impair the GAP activity of TBC-containing RabGAP proteins (*31*). Both GFP:TOPGAP and GFPTOPGAP^GAP^ successfully pulled down RFP:TIKI from *N. benthamiana* protein extracts but not the RFP:EV control (fig. S5B). Reciprocal co-IP experiments using RFP fusions of TOPGAP constructs with GFP:TIKI further validated the specific interaction between the effector and the RabGAP protein (fig. S5B). These results provide strong evidence that TIKI associates with TOPGAP in planta independent of the GAP activity of TOPGAP.

Across five independent experiments, we observed that overexpression of TOPGAP consistently enhanced *P. infestans* infection symptoms, notably increasing lesion size, compared to that of the EV control (Fig. 3B). Intriguingly, overexpression of the GAP mutant TOPGAP^GAP^ led to a significant reduction in infection lesion size relative to the EV control (Fig. 3B), suggesting a functional link between the GAP activity of TOPGAP and its role in plant immunity. To further substantiate the adverse effect of TOPGAP on immunity, we performed additional infection assays using the red fluorescent *P. infestans* strain 88069td. This approach allows for the direct quantification of pathogen biomass by measuring biotrophic hyphal growth using fluorescence microscopy. Parallel to our earlier infection assays (Fig. 3B), elevating TOPGAP levels significantly boosted *P. infestans* hyphal growth in three independent experiments (Fig. 3C). Conversely, the overexpression of the GAP mutant TOPGAP^GAP^ led to a marked decrease in pathogen hyphal growth compared to that of the EV control (Fig. 3C), indicating that the GAP activity of TOPGAP is crucial for facilitating disease susceptibility.

To complement the overexpression assays, we decided to down-regulate *TOPGAP* gene expression using RNA interference (RNAi). By performing BLAST analysis of TOPGAP in NbenBase (*32*), we identified a single full-length *TOPGAP* allele. Down-regulating the *TOPGAP* gene expression in *N. benthamiana* using a hairpin RNAi construct (RNAi:TOPGAP) (fig. S5C) significantly reduced *P. infestans* infection lesions compared to the silencing control, RNAi:GUS (Fig. 3D). In addition, silencing of TOPGAP also reduced *P. infestans* hyphal growth compared to the GUS silencing control (Fig. 3E). Collectively, these results further confirm the role of TOPGAP as a negative regulator of plant immunity. This notion is supported by the observed dominant negative phenotype of the TOPGAP^GAP^ mutant, which enhances resistance.

Given the regulatory role of RabGAPs in subcellular trafficking and our finding that TOPGAP negatively regulates plant immunity via its GAP function, we reasoned that TOPGAP might be controlling immune-related secretion. To test this hypothesis, we analyzed the potential impact of TOPGAP on defense-related secretion by monitoring the native levels of PR protein 1 (PR1) in the apoplast. We measured PR1 levels by using a specific antibody raised against it following overexpression of TOPGAP, TOPGAP^GAP^ mutant, or the GFP:EV control. We noted a marked decrease in PR1 secretion into the apoplast when TOPGAP was overexpressed, in contrast to the TOPGAP^GAP^ mutant or EV control overexpression (Fig. 3F). This outcome strongly supports the notion that TOPGAP subverts plant immunity by suppressing defense-related secretion.

### PiE354 co-opts TOPGAP to subvert plant immunity and defense-related secretion

**Fig. 4.**
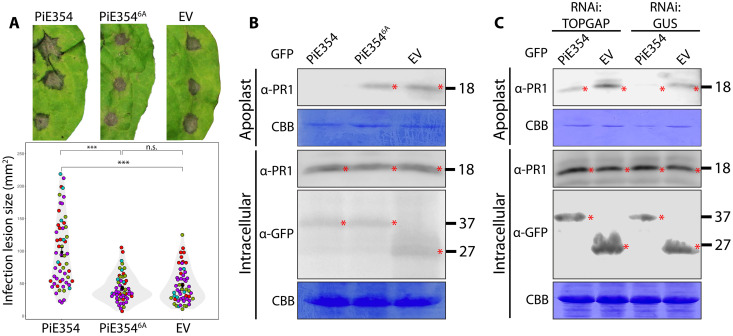
PiE354 co-opts TOPGAP to subvert plant immunity and defense-related secretion. (**A**) *N. benthamiana* leaves expressing PiE354, PiE354^6A^, or EV control were infected with WT *P. infestans*, and pathogen growth was calculated by measuring infection lesion size at 8 dpi. Each color represents an independent biological replicate. Each dot represents an infection spot. Statistical differences were analyzed by Mann-Whitney *U* test in R. Measurements were highly significant when ***P < 0.001. Detailed statistical analyses can be found in table S9. n.s., not significant. (**B** and **C**) For PR1 secretion assays, the infiltrated leaves were challenged with *P. infestans* extract at 3 dpi, and proteins were extracted from the apoplast and leaf tissue at 4 dpi and immunoblotted. (B) Western blot shows PiE354 suppresses antimicrobial PR1 secretion into the apoplast, dependent on the interaction with its host target TOPGAP. *N. benthamiana* leaves were infiltrated to express GFP:PiE354, GFP:PiE354^6A^ (mutant that lacks the capability to interact with TOPGAP), or GFP:EV. (C) Western blot shows that the ability of PiE354 to inhibit PR1 secretion is less pronounced when TOPGAP is silenced, compared to that under GUS-silenced control condition. *N. benthamiana* leaves were infiltrated to express RNAi:TOPGAP or RNAi:GUS control, with either PiE354 or EV control. Red asterisks show expected band sizes. Numbers on the right indicate kilodalton values.

To elucidate the functional relationship between PiE354 and TOPGAP, we first determined the extent to which PiE354 or its mutant PiE354^6A^, impaired in binding to its host target TOPGAP (Fig. 2D), influences susceptibility to *P. infestans.* We measured this through infection assays on *N. benthamiana* leaf patches that transiently express PiE354, PiE354^6A^, or EV control. In four independent infection assays, we observed a consistent increase in *P. infestans* infection lesion size in leaf patches expressing PiE354 compared to that in EV control (Fig. 4A). In contrast, leaf samples expressing PiE354^6A^ mutant did not exhibit any increase in infection lesion size relative to the EV control (Fig. 4A). These findings demonstrate that PiE354 enhances *P. infestans* virulence on plants and its ability to exacerbate *P. infestans* virulence is dependent on the residues required for interacting with TOPGAP.

We then investigated whether PiE354 similarly affects PR1 secretion in *N. benthamiana*, similar to the effects observed with TOPGAP overexpression (Fig. 3F). Heterologous expression of PiE354, but not the EV control, effectively suppressed PR1 secretion into the apoplast (Fig. 4B and fig. S6). In contrast, PiE354^6A^ mutant did not have any effect on PR1 secretion, behaving much like the EV control (Fig. 4B). This suggests that PiE354 mirrors the effects of TOPGAP overexpression, reinforcing the notion that it facilitates the GAP function of TOPGAP to hinder defense-related secretion (Fig. 3F).

Last, to ascertain the role of PiE354 in disrupting defense-related secretion through its interaction with TOPGAP, we conducted PR1 secretion assays in *TOPGAP*-silenced plants. As expected, in leaf patches with RNAi:GUS silencing control, PiE354 effectively reduced PR1 secretion into the apoplast, relative to the EV (Fig. 4C). Conversely, when *TOPGAP* was silenced, the capacity of PiE354 to inhibit PR1 secretion was less pronounced, although there was still a noticeable reduction in PR1 secretion compared to that of the EV (Fig. 4C). This is a reasonable outcome given the RNAi:TOPGAP construct does not fully deplete the TOPGAP transcripts (fig. S4C). These findings strongly suggest that the capacity of PiE354 to interfere with defense-related secretion and plant immunity is intricately linked to TOPGAP.

### Rab8a, a Rab GTPase that mediates defense-related secretion, is a GAP substrate of TOPGAP

We next focused on determining the cognate Rab GTPase partner of TOPGAP. Through an IP-MS interactome screen, we identified Rab8a as a candidate Rab substrate of TOPGAP (table S4). Given the previous findings by us and others that Rab8a plays a positive role in plant immunity against *P. infestans* and mediates PR1 secretion (*3*, *20*, *33*), we reasoned that Rab8a could be the cognate partner of TOPGAP.

**Fig. 5.**
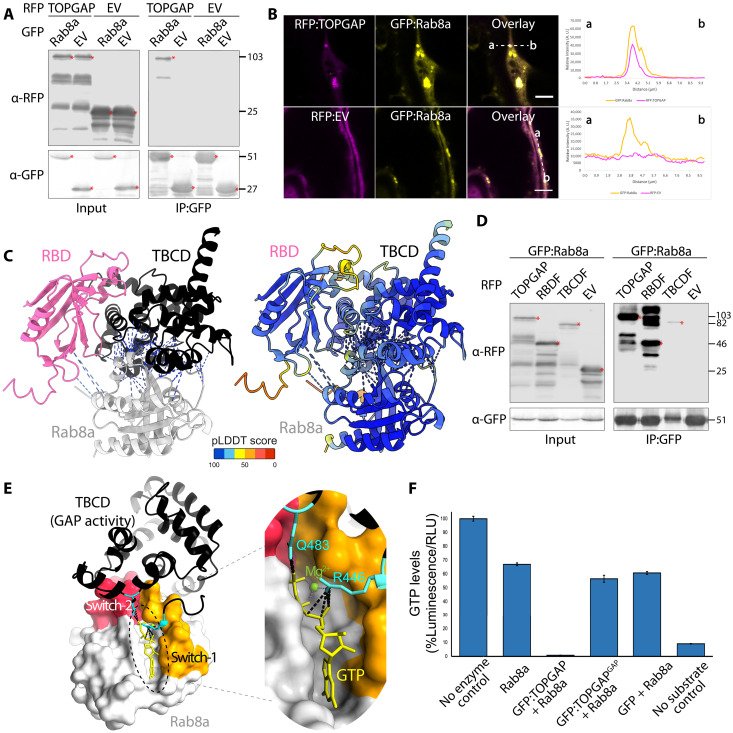
Rab8a is a GAP substrate of TOPGAP. (**A**) TOPGAP interacts with Rab8a in planta. RFP:TOPGAP was transiently co-expressed with GFP:Rab8a or the GFP:EV control. RFP:EV served as a control for RFP:TOPGAP. IPs were obtained with anti-GFP antibody. For Western blotting results, total protein extracts were immunoblotted, red asterisks indicate expected band sizes, and numbers on the right indicate kilodalton values. (**B**) Rab8a colocalizes with TOPGAP in puncta in planta. Confocal micrographs of *N. benthamiana* leaf epidermal cells transiently expressing RFP:TOPGAP or RFP:EV, with GFP:Rab8a. Presented images are single-plane images. Overlay panel transects correspond to line intensity plots showing relative fluorescence across the marked distance. Scale bars, 5 μm. (**C**) AF2-M modeling of Rab8a with individual RBD and TBCD of TOPGAP in complex. (Left) Rab8a interacts with both the RBD and TBCD of TOPGAP. (Right) The AF2-M model colors are based on the AF2-calculated prediction confidence score [predicted local distance difference test (pLDDT)] shown in the rectangular box. (**D**) Rab8a interacts with full-length TOPGAP, RBDF of TOPGAP, and TBCDF of TOPGAP. GFP:Rab8a was transiently co-expressed with either RFP:TOPGAP, RFP:RBDF, RFP:TBCDF, or RFP:EV control. IPs were obtained with anti-GFP antibody. (**E**) Predicted AF2-M model of Rab8a in complex with full-length TOPGAP, focusing on TBCDF of TOPGAP. The catalytic dual fingers of the TBCD, R446 and Q483 (cyan), are positioned across the GTP-binding pocket of Rab8a, flanked by switch-1 (pink) and switch-2 (orange) regions, and making contacts with the GTP molecule within the pocket. (**F**) TOPGAP stimulates the GTPase activity of Rab8a dependent on its GAP activity. A luciferase-based GTPase assay was used to quantify the amount of GTP levels. The bar graph illustrates the effect of TOPGAP, TOPGAP^GAP^, or GFP control on the GTPase activity of Rab8a across three technical repeats. No enzyme control does not contain Rab8a. “No substrate control” does not contain added GTP. RLU, relative light unit.

To corroborate our IP-MS findings, we set out to verify the interaction between Rab8a and TOPGAP through co-IPs. These assays confirmed that RFP:TOPGAP interacts with GFP:Rab8a but not with GFP:EV. In addition, RFP:EV control did not interact with GFP:Rab8a, indicating that TOPGAP specifically binds GFP:Rab8a (Fig. 5A). Our confocal microscopy analysis also showed that TOPGAP, but not the EV control, colocalizes with Rab8a in puncta (Fig. 5B).

The AF2-M prediction of the TOPGAP-Rab8a complex indicated interactions between Rab8a and both the RBD and TBCDs of TOPGAP, with the binding interface predominantly oriented toward the TBCD (fig. S7A). This bias could be due to the presence of available GAP-Rab crystal structures in the mammalian field and the higher sequence conservation of the TBCD compared to that of the RBD, resulting in higher confidence score models for the TBCD binding interface than the RBD interface. AF2-M modeling of Rab8a with individual RBD and TBCD of TOPGAP showed high-confidence interactions between each domain of TOPGAP and Rab8a (Fig. 5C). To determine whether Rab8a engages both the RBD and TBCD as suggested by the AF2-M models, we performed pull-down assays using Rab8a with the two TOPGAP fragments. Our co-IP results revealed that Rab8a interacts with both the RBD and TBCD fragments of TOPGAP (Fig. 5D), corroborating the AF2-M model predictions. However, it is difficult to discern whether Rab8a binds both domains simultaneously or alternates between them. To further characterize the binding mechanism between Rab8a and TOPGAP, we investigated whether their interaction is dependent on the GAP activity of TOPGAP. Co-IP and Western blot analysis showed that Rab8a interacts with both WT TOPGAP and its GAP mutant, but not with the EV control, indicating that Rab8a interacts with TOPGAP independently of the GAP function of TOPGAP (fig. S7B). Our confocal microscopy analysis also revealed that both TOPGAP and its GAP mutant, but not the EV control, colocalize with Rab8a (Fig. 5B and fig. S7C). These findings indicate that, while the RBD and TBCD of TOPGAP facilitate its association with Rab8a, the catalytic function of the GAP domain is dispensable for Rab8a binding.

Having validated the TOPGAP-Rab8a interaction, we next investigated the functional interplay between the two proteins, focusing on investigating whether Rab8a is a GAP substrate of TOPGAP. We first used AF2-M to visualize the interaction between TOPGAP and Rab8a, with a specific focus on the TBCD fragment that contains the GAP activity. AF2-M prediction of the Rab8a-TOPGAP complex indicated a high-confidence model in which the TBCD makes multiple contacts with the switch-1 and switch-2 regions of Rab8a, which are flanking the GTP-binding pocket of Rab8a and are crucial for regulating its GTP hydrolysis activity (fig. S7D). We then introduced GTP inside the Rab8a GTP pocket on the AF2-M model by using the crystal structure of human Rab8a bound to GTP (Protein Data Bank: 6WHE) as described before (*33*). The resulting model revealed that the catalytic dual fingers of the TBCD, specifically R446 and Q483, are favorably positioned to establish contacts with the GTP molecule within the Rab8a GTP-binding pocket (Fig. 5E). This high-confidence AF2-M model suggests a reasonable configuration of the GAP domain to catalyze the conversion of Rab8a-GTP to Rab8a-GDP.

To experimentally determine whether Rab8a is a substrate of TOPGAP, we conducted on beads GAP activity assays. We isolated total protein extracts from *N. benthamiana* leaves expressing GFP:TOPGAP, GFP:TOPGAP^GAP^ mutant, or GFP:EV control and concentrated them on GFP-trap beads via IP. Before assessing the activities of the immobilized constructs on beads, we confirmed the functionality of the GTPase activity of Rab8a purified from *Escherichia coli* (fig. S7E). Next, we incubated purified Rab8a alone or with GFP:TOPGAP, GFP:TOPGAP^GAP^, or the GFP:EV control. Rab8a alone induced a 30 to 35% reduction in GTP levels compared to the buffer with no enzyme control within 2 hours. Incubation of Rab8a with the affinity resin that pulled down GFP:TOPGAP completely depleted GTP levels (Fig. 5F). In contrast, incubation with GFP:TOPGAP^GAP^ or the EV control did not considerably alter GTP levels compared to that with Rab8a alone (Fig. 5F). These findings conclusively show that TOPGAP substantially enhances the GTP hydrolysis activity of Rab8a, acting as a canonical GAP, and this activity is reliant on the functional integrity of its TBCD.

### TOPGAP negatively regulates immunity by restricting Rab8a-mediated subcellular trafficking toward the cell surface

**Fig. 6.**
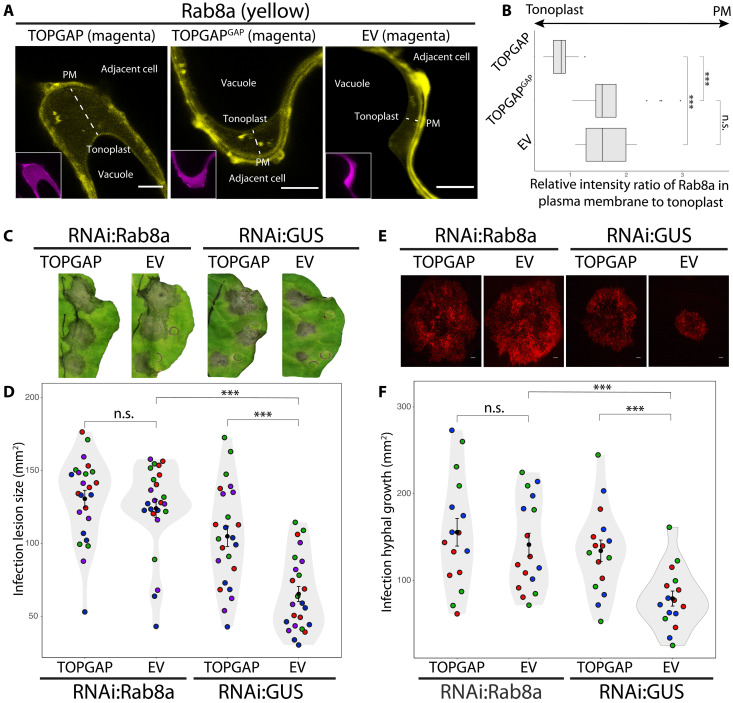
TOPGAP negatively regulates immunity by restricting Rab8a-mediated subcellular trafficking toward the cell surface. (**A** and **B**) TOPGAP diverts Rab8a localization from the plasma membrane to the tonoplast dependent on its GAP activity. (A) Confocal micrographs of *N. benthamiana* leaf epidermal cells transiently co-expressing either RFP:TOPGAP, RFP:TOPGAP^GAP^, or RFP:EV, with GFP:Rab8a. Presented images are single-plane images. Scale bars, 5 μm. (B) Box plot illustrates that TOPGAP expression significantly reduces the relative intensity ratio of Rab8a in plasma membrane to tonoplast compared to that in the EV control, while TOPGAP^GAP^ expression has no significant effect. Black dots denote outliers as indicated by ggplot2 in R. (**C**) The significant increase in *P. infestans* lesion size caused by TOPGAP expression is negated when Rab8a is silenced. RNAi:Rab8a or RNAi:GUS control was co-expressed with either TOPGAP or EV control in WT leaves. The agroinfiltrated leaves were infected with WT *P. infestans*, and pathogen growth was calculated by measuring infection lesion size at 7 dpi. (**D**) The significant increase in *P. infestans* hyphal growth caused by TOPGAP expression is negated when Rab8a is silenced. RNAi:Rab8a or RNAi:GUS control was co-expressed with either TOPGAP or EV control in WT leaves. The agroinfiltrated leaves were infected with tdTomato-expressing *P. infestans*, and pathogen growth was calculated by measuring hyphal growth using fluorescence stereomicroscope at 5 dpi. Scale bars, 10 μm. [(C) and (D)] Each color represents an independent biological replicate. Each dot represents the average of three infection spots on the same leaf. Statistical differences were analyzed by Student’s *t* test, or Mann-Whitney *U* test in R. Measurements were highly significant when ***P < 0.001. Detailed statistical analyses can be found in table S9.

Having determined the in vitro GAP activity of TOPGAP on Rab8a, we next sought to determine how TOPGAP regulates Rab8a functions in vivo. Our previous work showed that Rab8a localizes with a more pronounced signal at the plasma membrane compared to the vacuolar membrane (tonoplast), indicating a predominant Rab8a transport route toward the cell surface (*20*). To investigate whether TOPGAP modulates the subcellular localization of Rab8a, we used confocal microscopy to measure the relative intensity ratio of Rab8a in the plasma membrane to the tonoplast. This ratio was based on the relative fluorescence intensity levels on each membrane, calculated using ImageJ. Overexpression of RFP:TOPGAP resulted in a marked reduction of the plasma membrane levels of GFP:Rab8a with respect to the tonoplast, indicating the redistribution of GFP:Rab8a trafficking toward the vacuole (Fig. 6, A and B). In contrast, co-expression with either TOPGAP^GAP^ mutant or the EV control maintained the primary localization of GFP:Rab8a at the plasma membrane (Fig. 6, A and B), aligning with our previous findings (*20*). These results provide compelling evidence that TOPGAP regulates Rab8a-mediated trafficking from the cell surface to the vacuole, further affirming its role as a GAP for Rab8a. Consistent with prior research showing the role of Rab8a in PR1 release into the apoplast (*3*), we infer that TOPGAP hinders PR1 secretion (Fig. 3F) by redirecting Rab8a-mediated trafficking away from the cell surface.

Considering previous studies demonstrating the role of Rab8a in plant immunity, we hypothesized that the negative impact of TOPGAP on immunity might be due to its influence on Rab8a trafficking. To elucidate the interplay between TOPGAP and Rab8a in plant immunity, we silenced Rab8a using a hairpin RNAi construct (*20*) and examined whether TOPGAP can still suppress immunity. Consistent with the well-established defense roles of Rab8a against *P. infestans* (*3*, *20*), silencing Rab8a increased infection lesion sizes compared to the GUS-silencing control. In GUS-silenced plants, TOPGAP overexpression significantly enlarged infection lesions relative to the EV control, aligning with our findings of TOPGAP negatively affects immunity (Fig. 6C). Conversely, in Rab8a-silenced plants, TOPGAP overexpression did not significantly alter infection lesion sizes compared to that in the EV control (Fig. 6C). This pattern was also evident when measuring pathogen biomass in infection assays using the red fluorescent *P. infestans* strain 88069td (Fig. 6D). These results collectively indicate that the negative influence of TOPGAP on plant immunity is dependent on its regulation on Rab8a.

### PiE354 diverts Rab8a trafficking by hijacking the TOPGAP-Rab8a complex

**Fig. 7.**
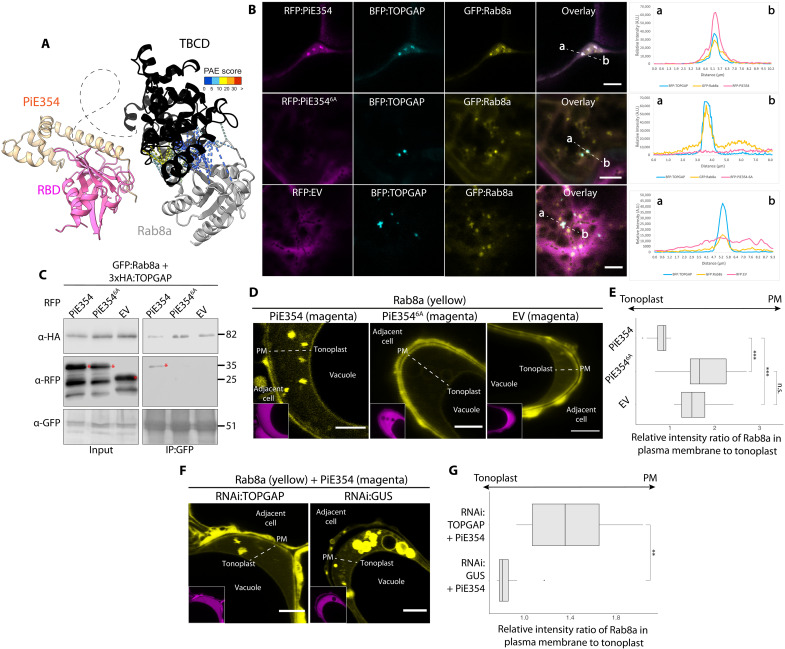
PiE354 targets the TOPGAP-Rab8a complex and re-routes Rab8a trafficking toward the vacuole. (**A**) AF2-M–predicted model of PiE354 in complex with the TOPGAP-Rab8a pair. The model indicates that in the presence of PiE354, Rab8a and the RBD of TOPGAP no longer interact. Bond colors reflect the AF2-calculated predicted aligned error (PAE) score. (**B**) Confocal micrographs of *N. benthamiana* leaf epidermal cells transiently co-expressing RFP:PiE354, RFP:PiE54^6A^, or RFP:EV, with BFP:TOPGAP and GFP:Rab8a. Overlay panel transects correspond to line intensity plots showing relative fluorescence across the marked distance. (**C**) PiE354 binds to the TOPGAP-Rab8a pair dependent on its interaction with TOPGAP. GFP:Rab8a and 3xHA:TOPGAP were transiently co-expressed with RFP:PiE354, RFP:PiE354^6A^, or RFP:EV. IPs were obtained with anti-GFP antibody. Red asterisks indicate expected band sizes. Numbers on the right indicate kilodalton values. (**D** and **E**) PiE354 diverts Rab8a localization from the plasma membrane to the tonoplast dependent on its interaction with TOPGAP. (D) Confocal micrographs of *N. benthamiana* cells transiently expressing RFP:PiE354, RFP:PiE354^6A^, or RFP:EV, with GFP:Rab8a. (E) Box plot illustrates that expression of EV control or PiE354^6A^ predominantly localizes Rab8a to the plasma membrane, while PiE354 redirects Rab8a primarily to the tonoplast. (**F** and **G**) Silencing *TOPGAP* nullifies the ability of PiE354 diverting Rab8a localization from plasma membrane to the tonoplast. (F) Confocal micrographs of *N. benthamiana* cells transiently expressing RNAi:TOPGAP, or RNAi:GUS control, with RFP:PiE354 and GFP:Rab8a. (G) Box plot illustrates that, under PiE354 expression, silencing *TOPGAP* leads to Rab8a predominantly localizing to the plasma membrane, while, in the GUS-silenced control condition, Rab8a primarily localizes to the tonoplast. All presented images are single-plane images. Scale bars, 5 μm. Black dots in box plots denote outliers indicated by ggplot2 in R. Statistical differences were analyzed by Mann-Whitney *U* test in R. Measurements were significant when **P < 0.01. Detailed statistical analyses can be found in table S9.

AF2-M modeling of the PiE354-Rab8a-TOPGAP complex reveals a compelling tripartite interaction (Fig. 7A and fig. S8), aligning with our protein-protein interaction assays (Figs. 2B and 5D). It appears that PiE354 alters the orientation of Rab8a toward the TBCD fragment harboring the GAP activity while associating with the RBD interface (Fig. 7A). This is supported by the loss of AF2-M–predicted contacts between RBD and Rab8a (Fig. 5C) when PiE354 is present (Fig. 7A).

To explore the potential tripartite interaction of PiE354, TOPGAP, and Rab8a, we first performed colocalization assays using confocal microscopy. In agreement with the AF2-M prediction of the complex mediated by TOPGAP, TOPGAP and Rab8a colocalized in punctate structures with PiE354 but not with the PiE354^6A^ mutant or the EV control (Fig. 7B). To gain further evidence that PiE354 can form a complex with TOPGAP and Rab8a, we next performed co-IPs using protein extracts from *N. benthamiana* leaves expressing GFP:Rab8a and 3xHA:TOPGAP with either RFP:PiE354, RFP:PiE354^6A^ mutant, or the RFP:EV control. GFP pull-down assays showed that GFP:Rab8a pulls down both 3xHA:TOPGAP and RFP:PiE354 but not the RFP:PiE354^6A^ mutant or RFP:EV (Fig. 7C). These results are in agreement with the predicted AF2-M models (Fig. 7A) and outputs of the colocalization assays (Fig. 7B), reinforcing the view that PiE354 targets the TOPGAP-Rab8a complex. Because Rab8a did not pull down the PiE354^6A^ mutant, which cannot bind TOPGAP, we conclude that Rab8a-PiE354 interaction is mediated by TOPGAP.

Building on these insights, we hypothesized that PiE354 targets TOPGAP to leverage its GAP activity for disrupting Rab8a-mediated trafficking. To determine whether PiE354 mirrors the effects of TOPGAP overexpression, specifically in redirecting Rab8a trafficking toward the vacuole, we conducted detailed observations using confocal microscopy. Quantitative image analysis showed that when co-expressed with PiE354, Rab8a predominantly localized to the tonoplast, diverging from its usual plasma membrane localization (*20*) observed with the PiE354^6A^ mutant or the EV control (Fig. 7, D and E, and fig. S9). This finding aligns with our hypothesis, suggesting that PiE354 mimics the effect of TOPGAP overexpression, promoting the deactivation of Rab8a by TOPGAP. This notion is further reinforced by our experiments on the diversion of Rab8a trafficking by PiE354 following the silencing of *TOPGAP*. Quantitative analysis of confocal micrographs from these experiments revealed that, in *TOPGAP*-silenced leaf patches but not in GUS-silenced control leaves, PiE354 was unable to redirect Rab8a toward the tonoplast (Fig. 7, F and G). These results, combined with the fact that the PiE354^6A^ mutant, which cannot bind TOPGAP, does not alter the trafficking of Rab8a, suggest that PiE354 potentiates the GAP activity of TOPGAP, ultimately diverting Rab8a trafficking from the cell surface to the vacuole. These findings are in line with our earlier results showing diminished PR1 secretion caused by the PiE354-TOPGAP complex (Fig. 4, B and C), showing that PiE354 engages TOPGAP to divert Rab8a-mediated trafficking critical for defense at the pathogen interface.

### A plant pathogen effector that redirects defense-related secretion by co-opting a key transport regulator

**Fig. 8.**
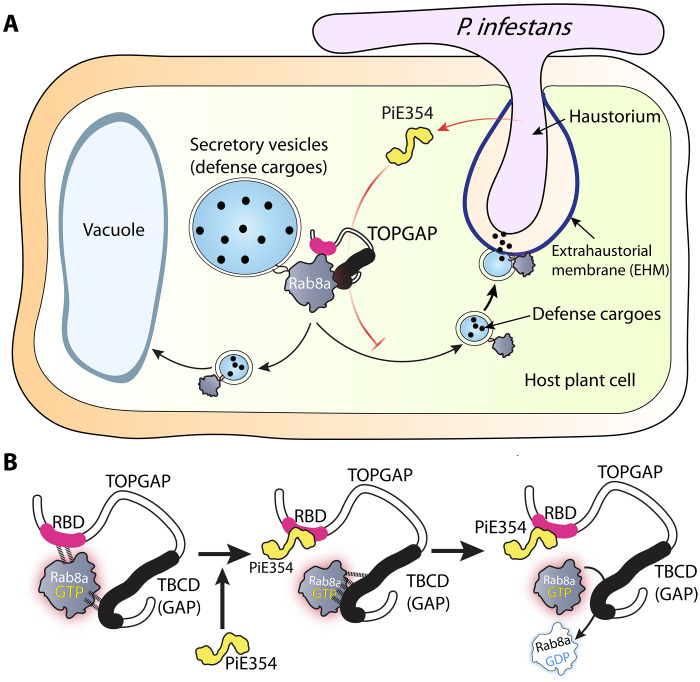
Summary models of the effector PiE354 co-opting the RabGAP TOPGAP and suppressing plant immunity by redirecting defense-related secretion. (**A**) Following penetration by *P. infestans* into the host plant cell, the effector PiE354 is secreted into the cytoplasm through its haustoria. Subsequently, PiE354 targets the host RabGAP protein TOPGAP, facilitating TOPGAP to deactivate its cognate RabGAP Rab8a. This leads to the redirection of Rab8a trafficking toward the vacuole instead of the plasma membrane, resulting in the suppression of plant immunity by inhibiting defense cargo secretion toward the pathogen interface. (**B**) In the absence of pathogen effectors, Rab8a interacts with both the RBD and TBCD of TOPGAP. Introduction of the effector PiE354, which binds to the RBD, causes a shift in the positioning of Rab8a toward the TBCD, where the GAP function is housed. Consequently, this facilitates the deactivation of Rab8a through increased GTP hydrolysis.

Our results suggest a model where PiE354, along with its homologs PiE355 and TIKI, harnesses the GAP function of TOPGAP on Rab8a, a key Rab GTPase involved in antimicrobial secretion. This process reroutes Rab8a-mediated antimicrobial secretion away from the pathogen interface and toward the vacuole, thereby hindering the ability of the plant to mount an effective immune response (Fig. 8A). We propose a mechanistic model in which PiE354 binds to the RBD of TOPGAP, propelling Rab8a toward the TBCD of TOPGAP, harboring the GAP function. This triggers an accelerated GTP hydrolysis on Rab8a, leading to its rapid disengagement from TOPGAP. This rapid turnover potentially facilitates the continuous and premature inactivation of nascent Rab8a-GTP molecules (Fig. 8B), perturbing their trafficking functions.

## DISCUSSION

Accumulating evidence points to host vesicle trafficking as a major hub targeted by pathogen effectors, although the underlying mechanisms are not well understood. Here, we uncovered an unprecedented immune subversion mechanism by which *Phytophthora* effectors, PiE354 and its homologs, remodel host membrane dynamics to prevent defense-related secretion through co-opting the host RabGAP protein TOPGAP. This manipulation effectively redirects defense-related trafficking governed by Rab8a, underscoring the pivotal but previously unknown role of TOPGAP in plant defense. Our findings provide a comprehensive molecular framework illustrating how pathogen effectors co-opt host regulatory components to transform the host-pathogen interface by subverting key immune pathways such as defense-related secretion.

### How does PiE354 co-opt TOPGAP to subvert Rab8a-mediated trafficking?

A key aspect of our findings is the elucidation of how PiE354 co-opts TOPGAP to manipulate Rab8a-mediated trafficking. We identified that DUF3548, reported to function as an RBD in the mammalian TBC-containing RabGAP protein RUTBC2 (*30*), fulfils a similar role in plant RabGAPs (Fig. 5, C and D). While some RabGAPs like TOPGAP are characterized by the presence of an RBD, the functional principles of the domain remained enigmatic. Our findings contribute to this understanding by revealing that PiE354 targets the RBD of TOPGAP, exploiting its GAP function on Rab8a (Fig. 7). This association hints at an intramolecular regulatory role for the RBD in modulating the GAP activity.

Building on this, our findings using AF2-M models (Fig. 5C) and co-IP experiments (Fig. 5D) indicate that Rab8a dynamically interacts with both the Rab-binding and TBCDs of TOPGAP. We propose that PiE354 binding to the RBD does not necessarily cause a premature release of GTP-bound Rab8a from TOPGAP, which would, otherwise, prevent Rab8a inactivation by TOPGAP. Instead, the docking of PiE354 at the RBD seems to guide Rab8a toward the TBCD, as indicated by structural predictions (Figs. 5C, 7A, and 8). This dynamic might negate any restrictive role of the RBD, such as hindering the access of Rab8a to the catalytic GAP interface, thereby boosting the conversion of Rab8a from its active GTP-bound state to its inactive GDP-bound state. However, further studies are needed to comprehensively understand the role of RBDs in TBC-containing RabGAP proteins across various species.

### *Phytophthora* effectors converge on Rab8a-mediated vesicle trafficking pathways

Our research highlights the significance of the Rab8 family of GTPases in plant immunity. This finding resonates with the known roles of the Rab8 family in polarized secretion in both animals and yeast (*34*) and its implication in plants for defense-related trafficking of PRRs to the plasma membrane (*3*). Consistent with the role of Rab8 in polarized secretion in other eukaryotes, our earlier work observed Rab8a-associated vesicles around *P. infestans* haustoria (*20*). This study further reveals that Rab8a-mediated trafficking is a key pathway in plant immunity, targeted by effectors from *P. infestans* and *P. palmivora* that co-opt TOPGAP, the cognate GAP of Rab8a. This aligns with findings that PexRD54, a *P. infestans* effector, redirects a subset of Rab8a toward autophagy (*20*) and RXLR242 from *P. capsici*, which hampers PR1 secretion by interacting with Rab8 members (*3*). It is likely that other pathogen effectors have evolved strategies to counter the functions of Rab8a-mediated trafficking, as highlighted by an earlier study, indicating the positive role of a Rab8 member in antibacterial immunity (*35*). These patterns align with the notion that effectors from the same or different pathogens converge on key immune pathways (*36*), emphasizing the critical role of Rab8a in plant defense. Although our study underscores the crucial role of TOPGAP in regulating Rab8a-mediated trafficking in plant defense, further research is needed to determine whether TOPGAP has regulatory roles across other Rab GTPases.

### PiE354-mediated remodeling of the pathogen interface

The PiE354-mediated subversion of host immunity exemplifies an elaborated strategy used by pathogen effectors to remodel the host-pathogen interface. By depleting Rab8a from the plasma membrane, PiE354 effectively hampers the secretion of PR1 into the apoplast (Fig. 4, B and C), highlighting a critical pathogen strategy in steering immune components away from the pathogen interface. This mechanism provides insight into the unique biochemistry of membrane interfaces at the pathogen incursion points, such as the formation of haustoria, indicative of a host susceptibility state.

In conclusion, our study illuminates the intricate interplay between plant vesicle trafficking and pathogen effectors, revealing an unprecedented immune evasion strategy to reconfigure pathogen interface. Specifically, we have shown that the effector PiE354 targets the plant RabGAP protein TOPGAP, effectively subverting defense-related trafficking governed by Rab8a. Our findings thus elucidate a sophisticated evolutionary adaptation by a pathogen effector, which capitalizes on the catalytic functionality of a host transport regulator to compromise innate immune responses.

## MATERIALS AND METHODS

### Molecular cloning

The molecular cloning of TOPGAP, TOPGAP^7M^, TOPGAP^GAP^, RBDF, TBCDF, PiE354, PiE354^6A^, and PiE355 was conducted using Gibson Assembly, following the methods described in previous works (*33*, *37*). Specifically, the vector backbone is a pK7WGF2 derivative domesticated for Gibson Assembly. The desired sequence for cloning was either manufactured as a synthetic fragment or amplified using designed primers. The fragments were then inserted into the vector using Gibson Assembly and then transformed into DH5α chemically competent *E. coli* through heat shock. These plasmids were subsequently amplified and extracted by PureYield Plasmid Miniprep System (Promega) and electroporated into *Agrobacterium tumefaciens* GV3101 electrocompetent cells. Sequencing was done by Eurofins. LB agar containing gentamicin and spectinomycin was used to grow constructs carrying pK7WGF2 plasmid. TIKI DNA was synthesized including an N-terminal FLAG tag and flanking attL1 and attL2 sites in pUC57, and FLAG-linker replaces the signal peptide. For the RNAi silencing construct of TOPGAP (RNAi:TOPGAP), an intron-containing hairpin RNA vector for RNAi in plants (pRNAi-GG) was used on the basis of Golden Gate cloning method described in a previous study (*38*). RNAi:TOPGAP targeted the region between 1790 and 2031 base pairs of *TOPGAP*. The target fragment (TOPGAP-silencing_synfrag) was synthesized and then inserted into the pRNAi-GG vector both in sense and antisense orientation, using the overhangs left by Bsa I cleavage. This resulted in the expression of a construct that folds back onto itself, forming the silencing hairpin structure. The subsequent steps of *E. coli* transformation, Miniprep, sequencing, and agrobacterium transformation were the same as those used for the overexpression constructs. LB agar containing gentamicin, kanamycin, and chloramphenicol was used to grow constructs carrying pRNAi-GG plasmid. All primers and synthetic fragments used in this study are detailed in table S5. All constructs used in this study are detailed in table S6.

### Plant material

*N. benthamiana* plants were cultivated in a controlled growth chamber at a temperature of 24°C, using a mixture of organic soil (3:1 ratio of Levington’s F2 with sand and Sinclair’s 2- to 5-mm vermiculite). The plants were exposed to high light intensity and subjected to a long day condition (16 hours of light and 8 hours of darkness photoperiod). The experiments were performed using plants that were 4 to 5 weeks old.

### *P. infestans* growth and infection assays

WT and tdTomato-expressing *Pytophthora infestans* 88069 isolates were cultivated on rye sucrose agar (RSA) medium in the dark at 18°C for a period of 10 to 15 days before harvesting zoospores (*39*). Zoospore solution was obtained by adding cold water at 4°C to the medium and then incubating it at 4°C in the dark for 90 min. For the infection assay, 10 μl of droplets of zoospore solution containing 50,000 spores/ml were applied to the abaxial side of agroinfiltrated leaves. The leaves were then kept in a humid environment. Daylight and fluorescent images were captured at 5 to 7 days postinoculation (dpi), and both lesion sizes and hyphal growth were measured and analyzed using ImageJ.

### Confocal laser scanning microscopy

The confocal microscopy analyses were conducted 3 days after agroinfiltration. To image the infiltrated leaf tissue, they were excised using a size 4 cork borer, live-mounted on glass slides, and submerged in wells of dH_2_O using Carolina observation gel (Carolina Biological). The imaging of the abaxial side of the leaf tissue was performed using either a Leica TCS SP8 inverted confocal microscope equipped with a 40× water immersion objective lens or a Leica STELLARIS 5 inverted confocal microscope equipped with a 63× water immersion objective lens. The laser excitations for GFP, RFP, and blue fluorescent protein (BFP) tags were argon at 488 nm (15%), diode-pumped solid-state (DPSS) at 561 nm, and diode at 405 nm, respectively. The emission ranges for GFP, RFP, and BFP tags were 495 to 550 nm, 570 to 620 nm, and 402 to 457 nm, respectively. To prevent spectral mixing from different fluorescent tags when imaging samples with multiple tags, sequential scanning between lines was applied. Confocal images, comprising both *Z*-stack and single-plane images, were analyzed using ImageJ.

### Fluorescence microscopy

Fluorescence microscopy was used to visualize the hyphal growth of *P. infestans* expressing tdTomato. The imaging setup consisted of a Leica MZ 16 F microscope coupled with the Leica DFC300 FX Digital Color Camera designed for fluorescence imaging. Infected leaf samples were positioned on a petri dish within the microscope imaging area. The imaging filter used was DsRed, with an excitation range spanning 510 to 560 nm.

### Structural and sequence analyses

The AF2-M was used via a Google Colab subscription, ColabFold v1.5.5. (*40*), adhering to the set guidelines (*41*). With the aid of the “align” command in UCSF ChimeraX (version 1.7), the AF2 predictions were superimposed onto known structures, and the confidence scores of the AF2 predictions were displayed using the local distance difference test (lDDT) scores on the lDDT-alpha-carbon atoms (Cα) metric (*42*). The scoring scale ranged from 0 to 100, with 100 indicating the highest confidence values. For sequence alignment, the MUSCLE algorithm was used (*43*), and the resulting alignments were visualized and color coded using ESPript 3.0 (*44*). Detailed information on the proteins and sequences used for AF2 can be found in table S7.

### Phylogenetic analyses

Homologs of TIKI were obtained using NCBI Blast (*45*). The full-length amino acid sequence of TIKI homologs was aligned using Clustal Omega version 1.2.2 (*46*). The amino acid replacement models were assessed, and the phylogenetic tree was generated using the phangorn package in R version 4.4.1 (*47*). The best amino acid replacement model “JTT” was selected by Bayesian information criterion. A maximum likelihood tree was generated with 100 bootstrap replicates, and the resultant tree was visualized with all bootstrap values using the ggtree package in R (*48*). The amino acid sequence similarities are calculated using Sequence Manipulation Suite (*49*).

### Agrobacterium-mediated transient gene expression in *N. benthamiana*

Agrobacterium-mediated transient gene expression was conducted through agroinfiltration, following the previously established method (*1*). *A. tumefaciens* carrying the desired plasmid was washed with water and then resuspended in agroinfiltration buffer [10 mM MES and 10 mM MgCl_2_ (pH 5.7)]. The optical density at 600 nm (OD_600_) of the bacterial suspension was measured using the BioPhotometer spectrophotometer (Eppendorf). Subsequently, the suspension was adjusted to the desired OD_600_ based on the construct and the specific experiment. The adjusted bacterial suspension was then infiltrated into 3- to 4-week-old *N. benthamiana* leaf tissue using a needleless 1-ml Plastipak syringe.

### RNA isolation, cDNA synthesis, and RT-PCR

To perform RNA extraction, 56 mg of leaf tissue was promptly frozen in liquid nitrogen. The RNA extraction process used the TRIzol RNA Isolation Reagent (Invitrogen), following the manufacturer’s guidelines. Subsequently, RNA concentration was quantified using NanoDrop Lite Spectrophotometer (Thermo Fisher Scientific). The extracted RNA (2 mg) underwent treatment with RQ1 ribonuclease-free deoxyribonuclease (Promega) before being used for cDNA synthesis with SuperScript IV Reverse Transcriptase (Invitrogen). The resulting cDNA was then amplified using Phusion High-Fidelity DNA Polymerase (New England Biolabs). Glyceraldehyde-3-phosphate dehydrogenase (GAPDH) level was used as the transcriptional control. The reverse transcription polymerase chain reaction (RT-PCR) for *TOPGAP* was performed using the primers TOPGAP_RTPCR_F and TOPGAP_RTPCR_R, while the RT-PCR for *GAPDH* was performed using the primers GAPDH_RTPCR_F and GAPDH_RTPCR_R. All primers used in this study are detailed in table S5.

### Co-IP and immunoblot analyses

Proteins were transiently expressed in *N. benthamiana* leaves through agroinfiltration, and the harvest took place 3 days after agroinfiltration. For Western blotting experiments, six leaf discs were excised using a size 4 cork borer (42 mg in total). Meanwhile, co-IP experiments used 2 g of leaf tissues. The procedures for protein extraction, purification, and immunoblot analysis followed the previously described protocols (*1*). The primary antibodies used included polyclonal anti-GFP produced in rabbit (ChromoTek), polyclonal anti-PR1 produced in rabbit (Agrisera), monoclonal anti-RFP produced in mouse (ChromoTek), and monoclonal anti-hemagglutinin (HA) produced in rat (ChromoTek). As for secondary antibodies, anti-rabbit antibody for horseradish peroxidase (HRP) detection and AP detection (Sigma-Aldrich), anti-mouse antibody for HRP detection (Sigma-Aldrich), and anti-rat antibody for HRP detection (Sigma-Aldrich) were used. Full-size Western blots are shown in fig. S10. Comprehensive information regarding the antibodies used is detailed in table S8.

### *P. infestans* extract preparation and injection

Mycelia obtained from *P. infestans* RSA plates were collected and suspended in 5 ml of water per petri dish. The suspension was vortexed for 1 min and subsequently heated at 95°C for 20 min. Then, the mixture was filtered through filter paper with a 5- to 13-μm pore size. The resulting filtrate underwent an additional filtration step using a syringe filter with a 0.45-μm pore size. This resultant solution was then administered to plants to serve as a pathogen-associated molecular pattern (PAMP) cocktail.

### Apoplastic washing fluid extraction

Apoplastic proteins were obtained following the previously described procedure (*50*). Detached and washed *N. benthamiana* leaves, which had undergone agroinfiltration, were rolled up and placed into a needleless syringe containing distilled water. Negative pressure was generated within the syringe to facilitate the infiltration of the entire leaves with water. Afterward, the infiltrated leaves were wrapped in parafilm, placed into a syringe with the plunger removed, and inserted into a Falcon tube. The entire setup was then centrifuged for 10 min at 1000*g* at 4°C. The apoplastic washing fluid accumulated at the bottom of the tube was collected and promptly frozen using liquid nitrogen. The remaining leaf tissue was gathered for subsequent immunoblotting analysis.

### Cell death assay

Cell death elicitors were introduced into the abaxial side of *N. benthamiana* leaves through agroinfiltration. Subsequently, at 2 to 4 dpi, the leaves were detached and subjected to imaging under both daylight and UV light conditions. The intensity of cell death was evaluated using a well-established seven-tiered cell death index (*51*).

### Rab8a purification

His-tagged Rab8a in Rosetta2 (DE3) pLysS strain *E. coli* was received from Y. Dagdas (Gregor Mendel Institute of Molecular Plant Biology, Vienna). In brief, *E. coli* transformed with Rab8a was grown to OD_600_ of 0.6 and induced overnight with 0.3 mM isopropyl-β-d-thiogalactopyranoside at 18°C. Harvested cells were frozen in −80°C until needed. Cells were resuspended in 100 mM sodium phosphate (pH 7.2), 300 mM NaCl, and 50 mM imidazole (buffer A) with an EDTA-free protease inhibitor tablet (cOmplete, Roche). Following cell disruption [CF Cell Disrupter (Constant Systems Ltd.) at 27 kpsi, 4°C, for three times] and ultracentrifugation, clear lysate was applied to a 5-ml HisTrap HP (Cytiva) using a peristaltic pump (P1, Cytiva). Elutions were performed using an imidazole gradient (buffer A and buffer A + 500 mM imidazole) applied by an ÄKTA pure protein purification system (Cytiva). Fractions were analyzed with SDS–polyacrylamide gel electrophoresis and eluted Rab8a was pooled and concentrated using an Amicon Ultra 30-kDa centrifugation filter. Rab8a was dialysed into 100 mM sodium phosphate (pH 7.2) and 150 mM NaCl buffer and stored at −80°C. Using purified Rab8a above the concentration of 0.1 mg/ml led to a reduction in GTP levels, verifying the functionality of the purified Rab8a (fig. S7E). We decide to carry out subsequent GTPase activity assays with Rab8a (0.2 mg/ml) because this concentration gave a robust and clear GTPase readout.

### GTPase activity assay

To investigate the impact of proteins of interest on the GTPase activity of Rab8a, we used a luciferase-based GTPase assay using the GTPase-Glo Assay Kit (Promega). The assay was conducted following the guidelines provided by the manufacturer. Specifically, a mastermix of 2× GTP-GAP solution was prepared, containing 10 μM GTP and 1 mM DTT in GTPase/GAP buffer. In each well of the microplate, 12.5 μl of Rab8a (0.4 mg/ml) was added, which was diluted in the buffer provided. The GTPase reaction was initiated by adding 12.5 μl of the 2× GTP-GAP solution to each well. The reaction was incubated for 120 min at room temperature with continuous shaking. Upon completion of the GTPase reaction, 25 μl of reconstituted GTPase-Glo Reagent was introduced to convert the unhydrolyzed GTP to ATP. The plate was then incubated for 30 min at room temperature with shaking. Subsequently, 50 μl of the Detection Reagent was added to all the wells, and they were incubated for 10 min at room temperature. Last, luminescence was measured using CLARIOstar Plus plate reader.

### Image processing and data analysis

The confocal microscopy images were processed using Leica LAS X software and ImageJ. Depending on the specific experiment, the confocal images could be either single-plane images or *Z*-stack images, and this information is provided in the figure legends. Image analysis and quantification for cell death and infection assay experiments were performed using ImageJ. For data representation, violin plots and box plots were created using ggplot2 in R (*52*), while bar graphs were generated using Microsoft Excel. To assess statistical differences, a range of tests, including Student’s *t* test and Mann-Whitney *U* test, were conducted on the basis of statistical normality and variance. Measurements were deemed significant when *P < 0.05 and **P < 0.01 and highly significant when ***P < 0.001. Detailed information regarding all the statistical calculations can be found in table S9.

### Accession numbers/identifiers

TOPGAP (Nbe.v1.s00100g29830), TIKI (PLTG_0964243; table S1), PiE354 (PITG_04354; table S1), and PiE355 (PITG_04355; table S1).

## Supplementary Materials

### The PDF file includes:

Figs. S1 to S10 Tables S5 and S6 Legends for tables S1 to S4 and S7 to S9 References
