# Supplementary material for: A pathogen effector co-opts a host RabGAP protein to remodel pathogen interface and subvert defense-related secretion
Source: Sci Adv. 2024 Oct 4;10(40):eado9516. doi: 10.1126/sciadv.ado9516 (PMC11451530; doi:10.1126/sciadv.ado9516)
Supplement: Supplementary file 1 — Figs. S1 to S10 Tables S5 and S6 Legends for tables S1 to S4 and S7 to S9 References [file sciadv.ado9516_sm.pdf]

Science Advances
AAAS

Supplementary Materials for

A pathogen effector co-opts a host RabGAP protein to remodel pathogen interface and subvert defense-related secretion

Enoch Lok Him Yuen *et al.*

Corresponding author: Tolga Osman Bozkurt, o.bozkurt@imperial.ac.uk;
Sebastian Schornack, sebastian.schornack@slcu.cam.ac.uk

Sci. Adv. **10**, eado9516 (2024)
DOI: 10.1126/sciadv.ado9516

The PDF file includes:

Figs. S1 to S10
Tables S5 and S6
Legends for tables S1 to S4 and S7 to S9
References

Other Supplementary Material for this manuscript includes the following:

Tables S1 to S4 and S7 to S9

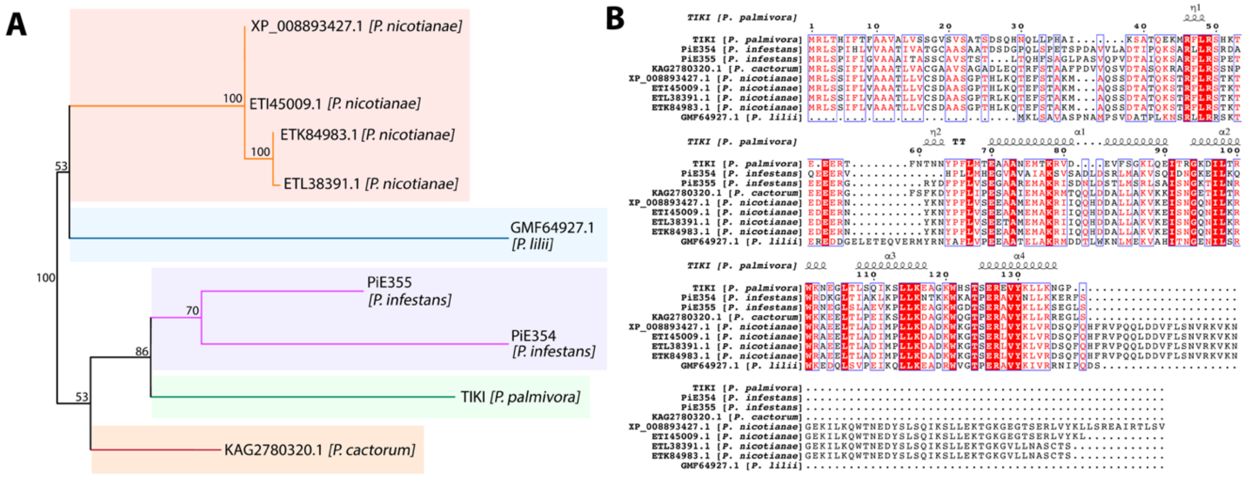

Fig. S1. TIKI belongs to an RXLR family of effectors and is conserved among the genus *Phytophthora*. (A) Phylogenetic tree of the conserved TIKI effectors. The phylogenetic tree was created using the full sequences of conserved TIKI effectors from the *Phytophthora* genus, including the pathogens *P. nicotianae, P. lilii, P. infestans, P. palmivora,* and *P. cactorum*. The tree was constructed using the ggtree package in R. (B) Pairwise amino acid sequence alignment of the conserved *Phytophthora* effectors as illustrated in Fig. S1A. Full sequences of the effectors were used for the alignments, which were obtained using the MUSCLE algorithm and visualized using ESPript 3.0 (*45*). The alignment is color-coded: red boxes indicate strictly identical residues, while blue frames indicate similar residues. The squiggle symbols above the sequences denote α-helices.

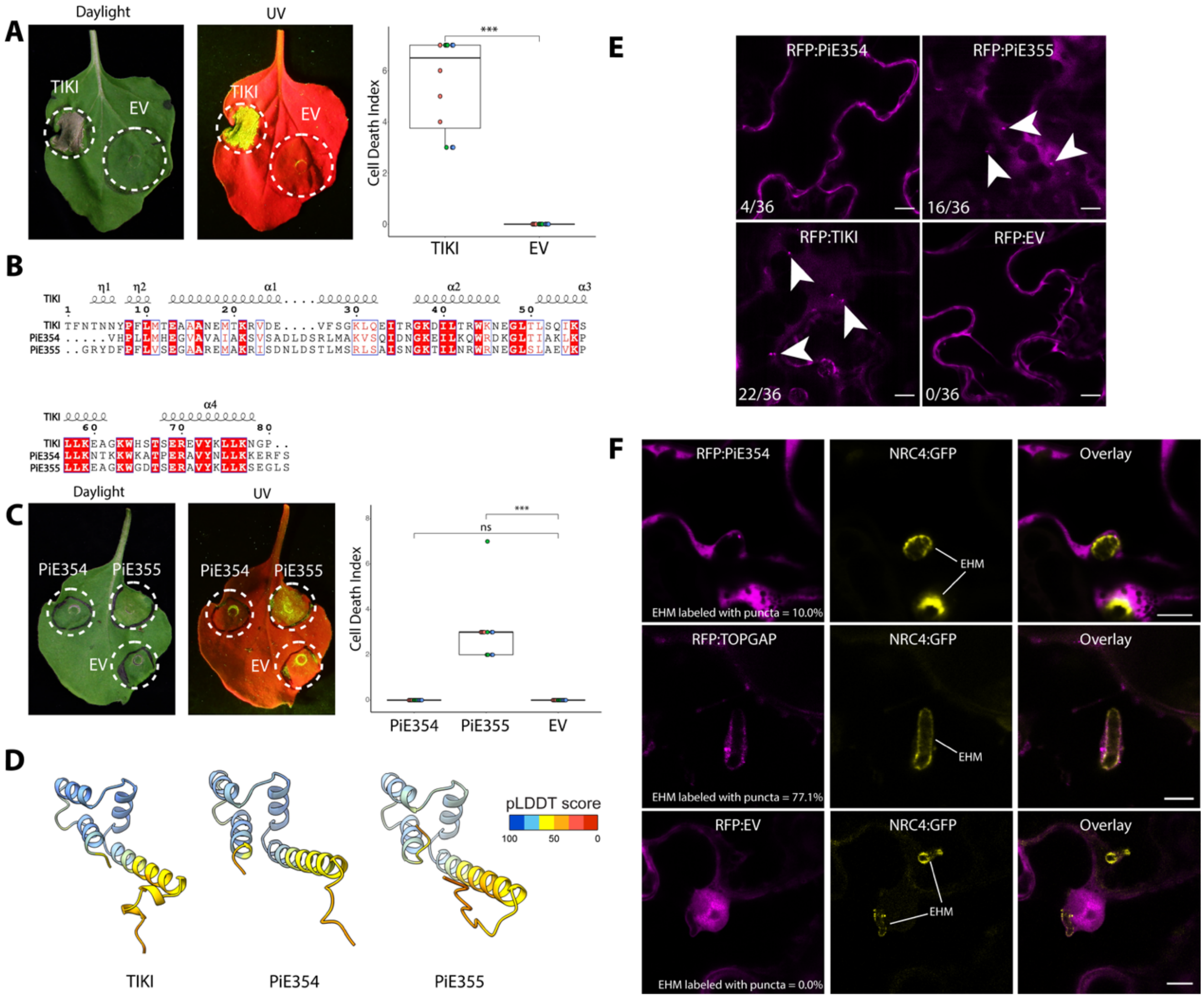

Fig. S2. PiE354 does not elicit a cell death response, and occasionally forms punctate structures *in planta*. (A) Expression of TIKI causes cell death. Representative *N. benthamiana* leaves infiltrated with TIKI or EV. Daylight and UV images were taken at 4 dpi, and cell death was scored at 4 dpi. Box and dot plot showing TIKI expression causes cell death in plants (5.5, N = 12), while EV control does not (0.0, N = 12). (B) Pairwise amino acid sequence alignment of the effector domains of the *Phytophthora* effectors TIKI, PiE354 and PiE355. Alignments were obtained using the MUSCLE algorithm and were visualized and color-coded via ESPript 3.0 (*45*). The alignment is color-coded: red boxes indicate strictly identical residues, while blue frames indicate similar residues. The squiggle symbols above the sequences denote α-helices. (C) Expression of PiE355, but not PiE354, causes cell death. Representative *N. benthamiana* leaves infiltrated with PiE354, PiE355, or EV. Daylight and UV images were taken at 4 dpi, and cell death was scored at 4 dpi. Box and dot plot showing PiE355 expression causes cell death in plants (3.0, N = 12), while PiE354 expression (0.0, N = 12) and EV expression (0.0, N = 12) do not. (D) AF2 structures of the effectors TIKI from *P. palmivora*, and PiE354 & PiE355 from *P. infestans*. The colors of TIKI, PiE354 and PiE355 are based on the AF2-calculated prediction confidence score (pLDDT) as indicated in the rectangular box. (E) PiE354 forms punctate structures less frequently than PiE355 and TIKI. Confocal micrographs of *N. benthamiana* leaf epidermal cells

transiently expressing RFP:PiE354, RFP:PiE355, RFP:TIKI, or RFP:EV control. For RFP:PiE354, 4 out of 36 images contain punctate structures. For RFP:PiE355, 16 out of 36 images contain punctate structures. For RFP:TIKI, 22 out of 36 images contain punctate structures. For RFP:EV, none of the 36 images contain punctate structures. White arrows indicate punctate structures. Presented images are single plane images. Scale bars, 5 µm. (F) Confocal micrographs of *N. benthamiana* leaf epidermal cells transiently expressing RFP:PiE354, RFP:TOPGAP, or RFP:EV control, along with NRC4:GFP. NRC4:GFP acts as a haustorial marker (*54*). The leaves were infected with WT *P. infestans* spores at 6 hpi and imaged at 3 dpi. Presented images are single plane images. Scale bars, 5 µm. All statistical differences were analyzed by Mann-Whitney U test in R. Measurements were highly significant when $p < 0.001$ (***).

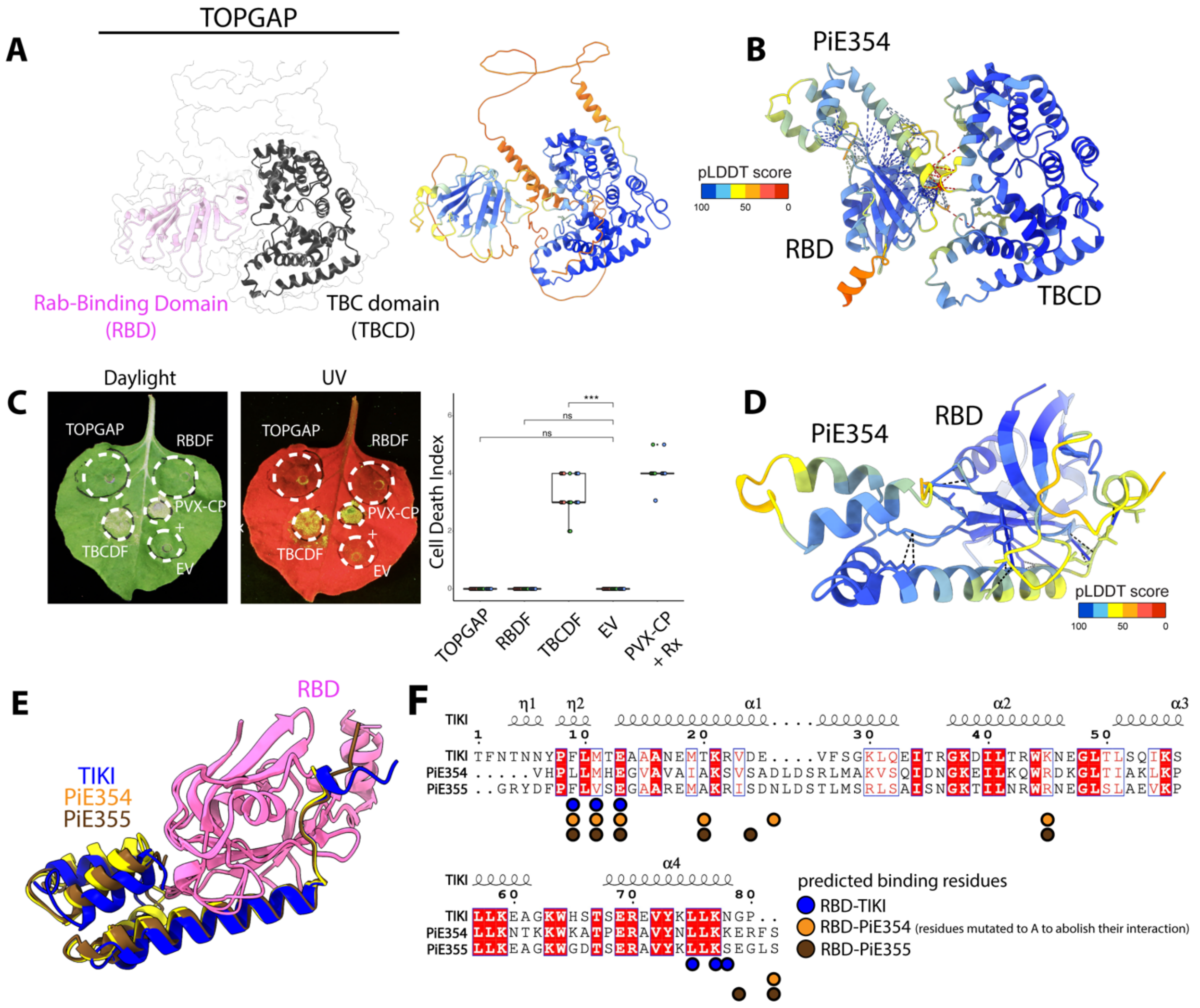

Fig. S3. PiE354 targets the N-terminal RBD fragment of TOPGAP. (A) AF2 model of TOPGAP architecture. (Left panel) A Rab-binding domain (RBD) is located at the N-terminal, and a TBC domain (TBCD) is located at the C-terminal. (Right panel) The colors of the AF2 model of TOPGAP are based on the AF2-calculated prediction confidence score (pLDDT) as indicated in the rectangular box. (B) AF2-M-predicted model of PiE354 targeting TOPGAP. The colors of the PiE354-TOPGAP AF2-M model are based on the AF2-calculated prediction confidence score (pLDDT) as indicated in the rectangular box. (C) Expression of TBCDF elicits cell death activity. Representative *N. benthamiana* leaves infiltrated with TOPGAP, RBDF, TBCDF or EV control. PVX-CP and Rx were co-infiltrated as a positive control for cell death activity. Daylight and UV images were taken at 4 dpi, and cell death was scored at 4 dpi. Box and dot plot showing TBCDF expression causes cell death in plants (3.3, N = 20) while TOPGAP (0.0, N = 20), RBDF (0.0, N = 20) and EV control expression (0.0, N = 20) do not. Statistical differences were analyzed by Mann-Whitney U test in R. Measurements were highly significant when $p<0.001$ (***). (D) AF2-M-predicted model of PiE354 targeting the RBD of TOPGAP. The colors of the PiE354-RBD AF2-M model are based on the AF2-calculated prediction confidence score (pLDDT) as indicated in the rectangular box. (E) Structural alignment of the predicted complexes RBD-PiE354, RBD-PiE355, and RBD-TIKI. Structural predictions were obtained via AF2. The model indicates

conservation of their binding interfaces, with an RMSD value of 0.4 between RBD-PiE354 and RBD-TIKI, and an RMSD of 0.483 between RBD-PiE354 and RBD-PiE355. (F) Pairwise amino acid sequence alignment of the effector domains of TIKI, PiE354, and PiE355. Alignments were obtained using the MUSCLE algorithm and were visualized and color-coded via ESPript 3.0 (*45*). The AF2-M predicted RBD-interacting residues on TIKI, PiE354, and PiE355 are labeled with blue, orange, and brown dots, respectively. The residues labelled as orange dots were mutated to alanine to abolish the *in planta* interaction between TOPGAP and PiE354 (Fig. 2D).

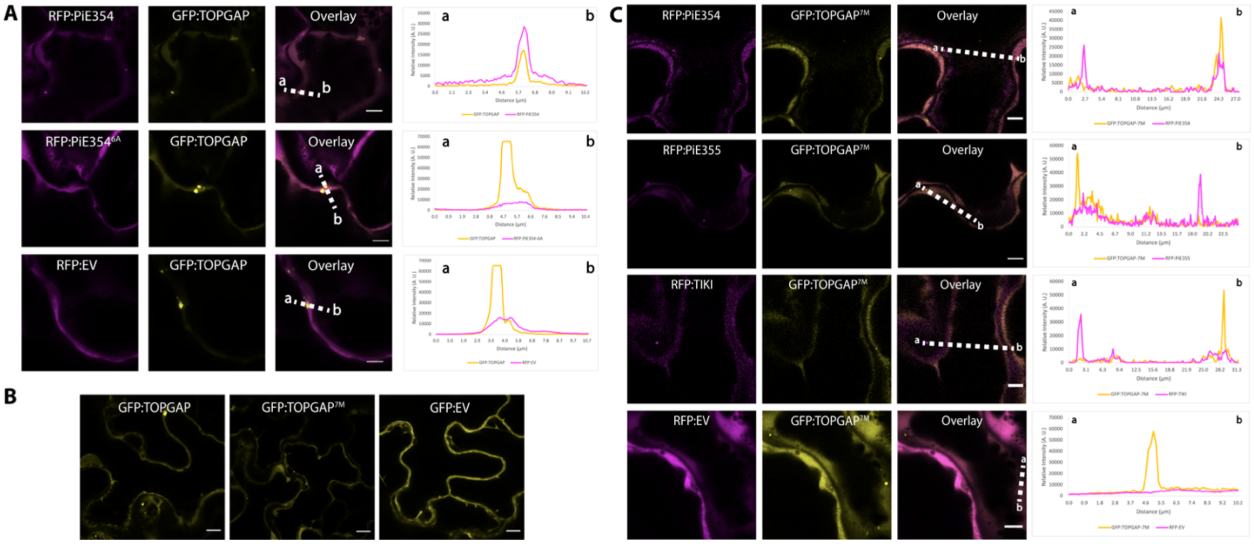

Fig. S4. PiE354 colocalizes with TOPGAP through 6 key residues. (A) Confocal micrographs of *N. benthamiana* leaf epidermal cells transiently expressing either RFP:PiE354 (1st row), RFP:PiE354^{6A} (2nd row), or RFP:EV (3rd row), with GFP:TOPGAP. (B) TOPGAP and TOPGAP^{7M} show cytoplasmic localization with puncta formation. Confocal micrographs of *N. benthamiana* leaf epidermal cells transiently expressing GFP:TOPGAP, GFP:TOPGAP^{7M}, or GFP:EV control. (C) TOPGAP^{7M} does not colocalize with the effectors PiE354, PiE355 and TIKI in puncta. Confocal micrographs of *N. benthamiana* leaf epidermal cells transiently co-expressing GFP:TOPGAP^{7M} with either RFP:PiE354 (1st row), RFP:PiE355 (2nd row), RFP:TIKI (3rd row), or RFP:EV control (4th row). All presented confocal images are single plane images. Overlay panel transects correspond to line intensity plots showing relative fluorescence across the marked distance. Scale bars, 5 µm.

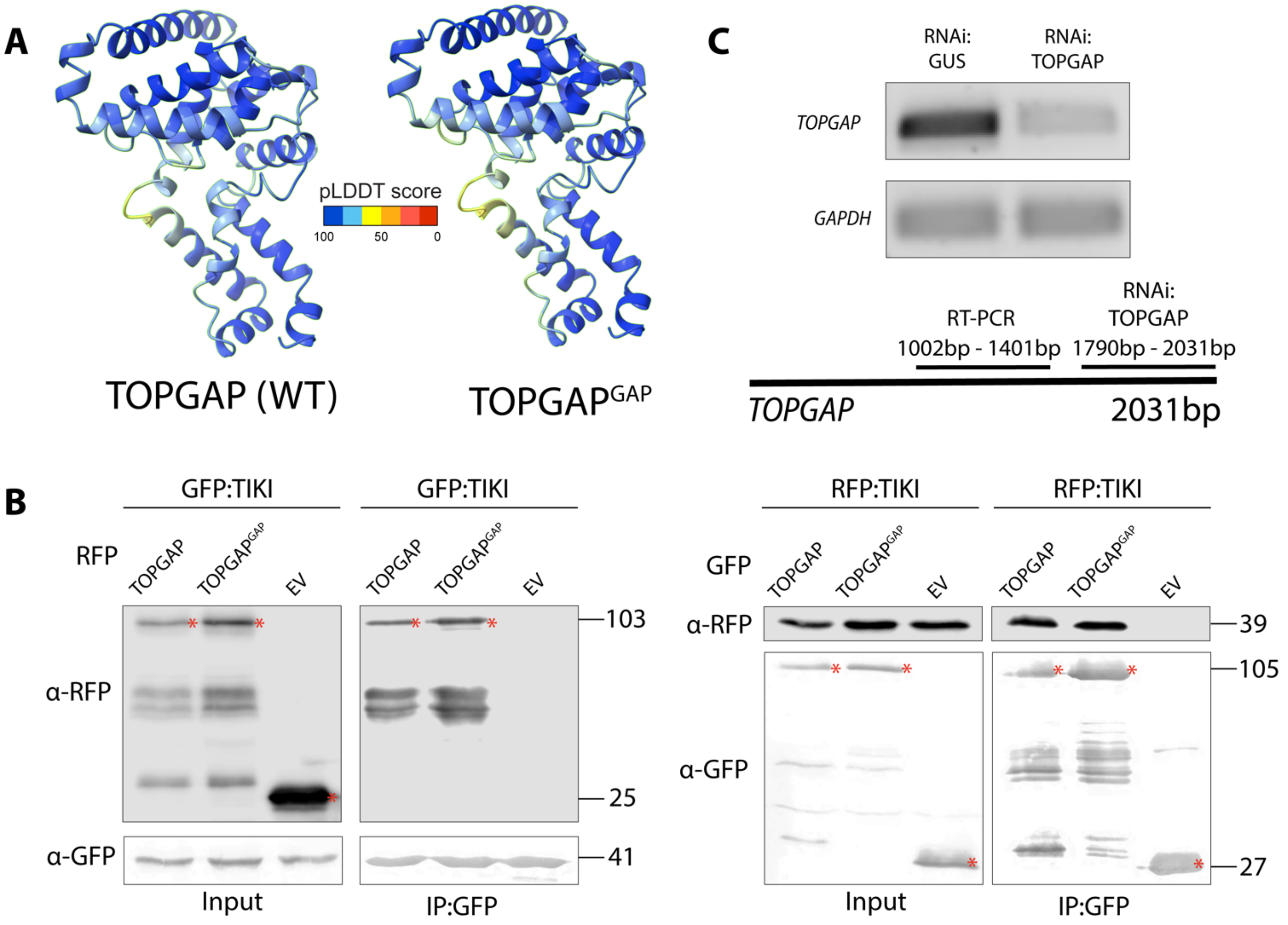

Fig. S5. TOPGAP interacts with TIKI independent of its GAP function. (A) AF2-predicted structures of TOPGAP and its GAP mutant TOPGAP^{GAP}. The colors of TOPGAP and TOPGAP^{GAP} are based on the AF2-calculated prediction confidence score (pLDDT) as indicated in the rectangular box. (B) TIKI interacts with TOPGAP *in planta* independent of the GAP function of TOPGAP. (Left panel) GFP:TIKI was transiently co-expressed with either RFP:TOPGAP, RFP:TOPGAP^{GAP}, or RFP:EV. IPs were obtained with anti-GFP antibody. (Right panel) RFP:TIKI was transiently co-expressed with either GFP:TOPGAP, GFP:TOPGAP^{GAP}, or GFP:EV. IPs were obtained with anti-GFP antibody. Total protein extracts were immunoblotted. Red asterisks indicate expected band sizes. Numbers on the right indicate kDa values. (C) Validation of *TOPGAP* silencing by RNAi:TOPGAP. Constructs carrying hairpin plasmids (pRNAi-GG) targeting TOPGAP or the GUS reporter gene were infiltrated to *N. benthamiana* leaves. The expression levels of the targeted genes were assessed via RT-PCR at 3 days post silencing. RT-PCR, employing primers TOPGAP_RTPCR_F and TOPGAP_RTPCR_R, confirmed efficient gene silencing of TOPGAP using the RNAi:TOPGAP construct. Glyceraldehyde 3-phosphate dehydrogenase (GAPDH) served as the internal control, using primers GAPDH_RTPCR_F and GAPDH_RTPCR_R for assessment. The cDNA was synthesized using total RNA.

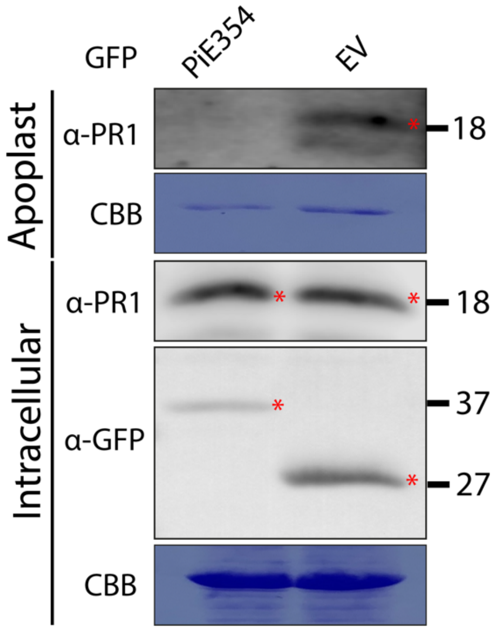

Fig. S6. PiE354 disrupts antimicrobial PR1 secretion into the apoplast. To conduct PR1 secretion assays, the infiltrated leaves were challenged with *P. infestans* extract at 3 dpi and proteins were extracted from the apoplast and leaf tissue at 4 dpi and immunoblotted. Western blot shows PiE354 disrupts antimicrobial PR1 secretion into the apoplast. *N. benthamiana* leaves were infiltrated to express GFP:PiE354, or GFP:EV. Red asterisks show expected band sizes. Numbers on the right indicate kDa values.

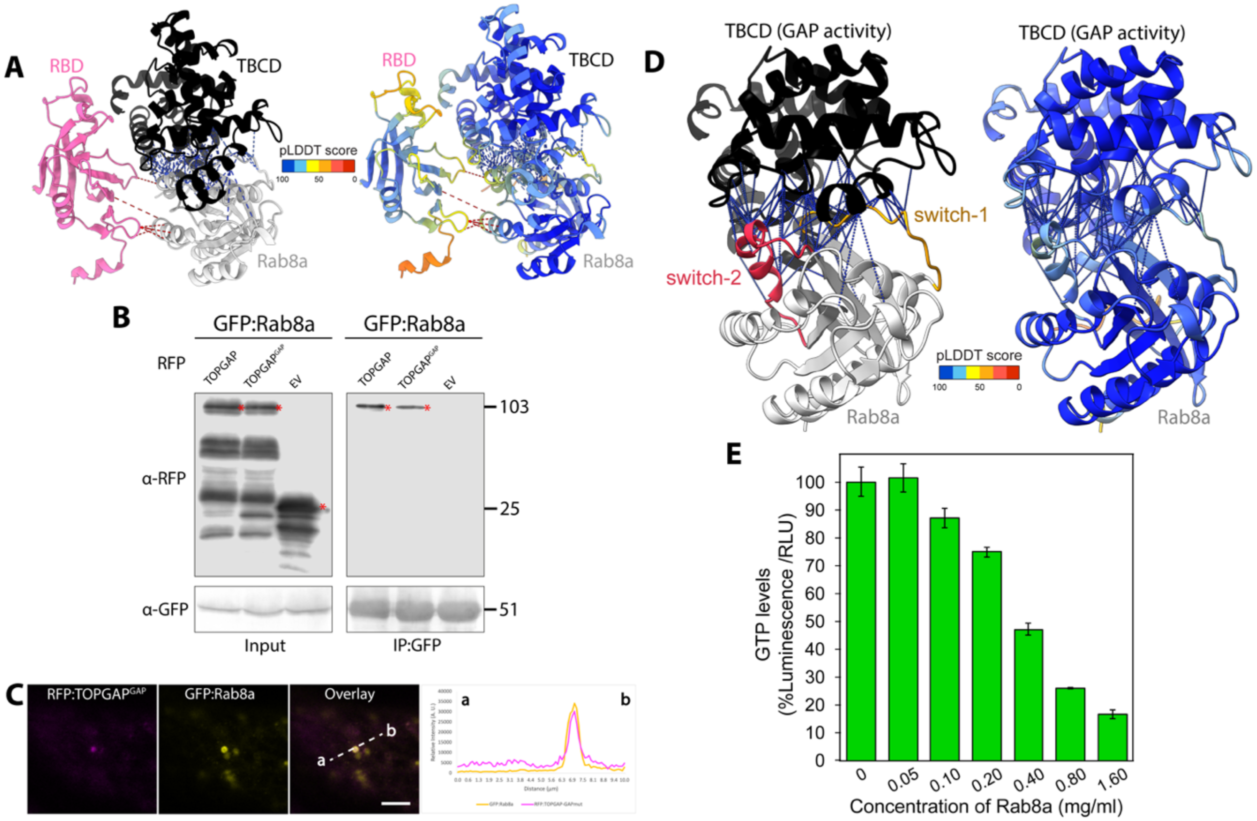

Fig. S7. Rab8a is a GAP substrate of TOPGAP. (A) AF2-M-predicted model of full length TOPGAP and Rab8a in complex. (Left panel) Rab8a interacts with both the RBD fragment (RBDF) and TBCD fragment (TBCDF) of TOPGAP. (Right panel) The colors of full length TOPGAP-Rab8a AF2-M model are based on the AF2-calculated prediction confidence score (pLDDT) as indicated in the rectangular box. (B) Rab8a interacts with TOPGAP *in planta* independent of the GAP activity of TOPGAP. GFP:Rab8a was transiently co-expressed with either RFP:TOPGAP, RFP:TOPGAP^{GAP}, or RFP:EV. IPs were obtained with anti-GFP antibody. Total protein extracts were immunoblotted. Red asterisks indicate expected band sizes. Numbers on the right indicate kDa values. (C) Rab8a colocalizes with TOPGAP in puncta independent of the GAP activity of TOPGAP. Confocal micrographs of *N. benthamiana* leaf epidermal cells transiently expressing RFP:TOPGAP^{GAP} with GFP:Rab8a. Presented images are single plane images. Overlay panel transects correspond to line intensity plots showing relative fluorescence across the marked distance. Scale bars, 5 µm. (D) AF2-M-predicted model of Rab8a in a complex with the TBCD of TOPGAP. (Left panel) TBCD, which is crucial for the GAP activity of TOPGAP, makes multiple contacts with the switch-1 and switch-2 regions of Rab8a that regulate GTP hydrolysis activity. Switch-1 and switch-2 regions that are flanking the GTP binding pocket are colored bronze and red respectively. (Right panel) The colors of TBCDF-Rab8a AF2-M model are based on the AF2-calculated prediction confidence score (pLDDT) as indicated in the rectangular box. (E) Bar graph depicting the impact of varying concentrations of Rab8a on intrinsic GTPase activity. Serially diluted Rab8a in GTPase/GAP Buffer was combined with 2X GTP solution containing 10 µM GTP and 1 mM DTT, resulting in initial reaction mixtures with Rab8a concentrations ranging from 0 to 1.60 mg/ml. These mixtures were then incubated at 25°C for 120 minutes as per manufacturer's guidelines. An equal volume of reconstituted GTPase-Glo™ Reagent was added

to the solutions to cease the GTP hydrolysis reaction, and the mixture was incubated for 30 minutes at room temperature. Following the incubation, the detection buffer was added, and luminescence levels were recorded, which correspond to the quantity of unhydrolyzed GTP remaining in the solution after the GTPase reaction.

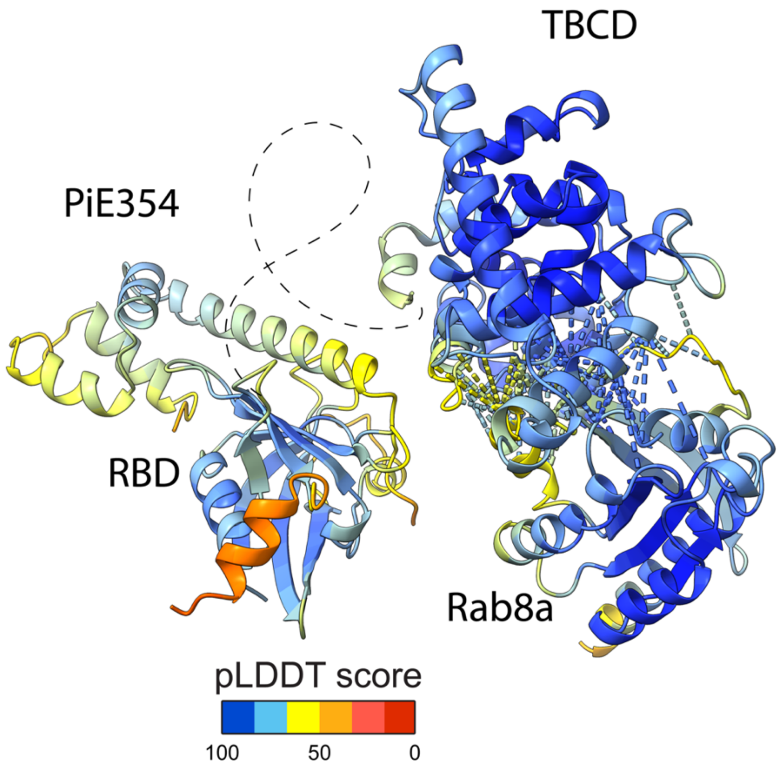

Fig. S8. AF2-M-predicted model of PiE354 in complex with the TOPGAP-Rab8a pair. The colors of PiE354-TOPGAP-Rab8a AF2-M model are based on the AF2-calculated prediction confidence score (pLDDT) as indicated in the rectangular box.

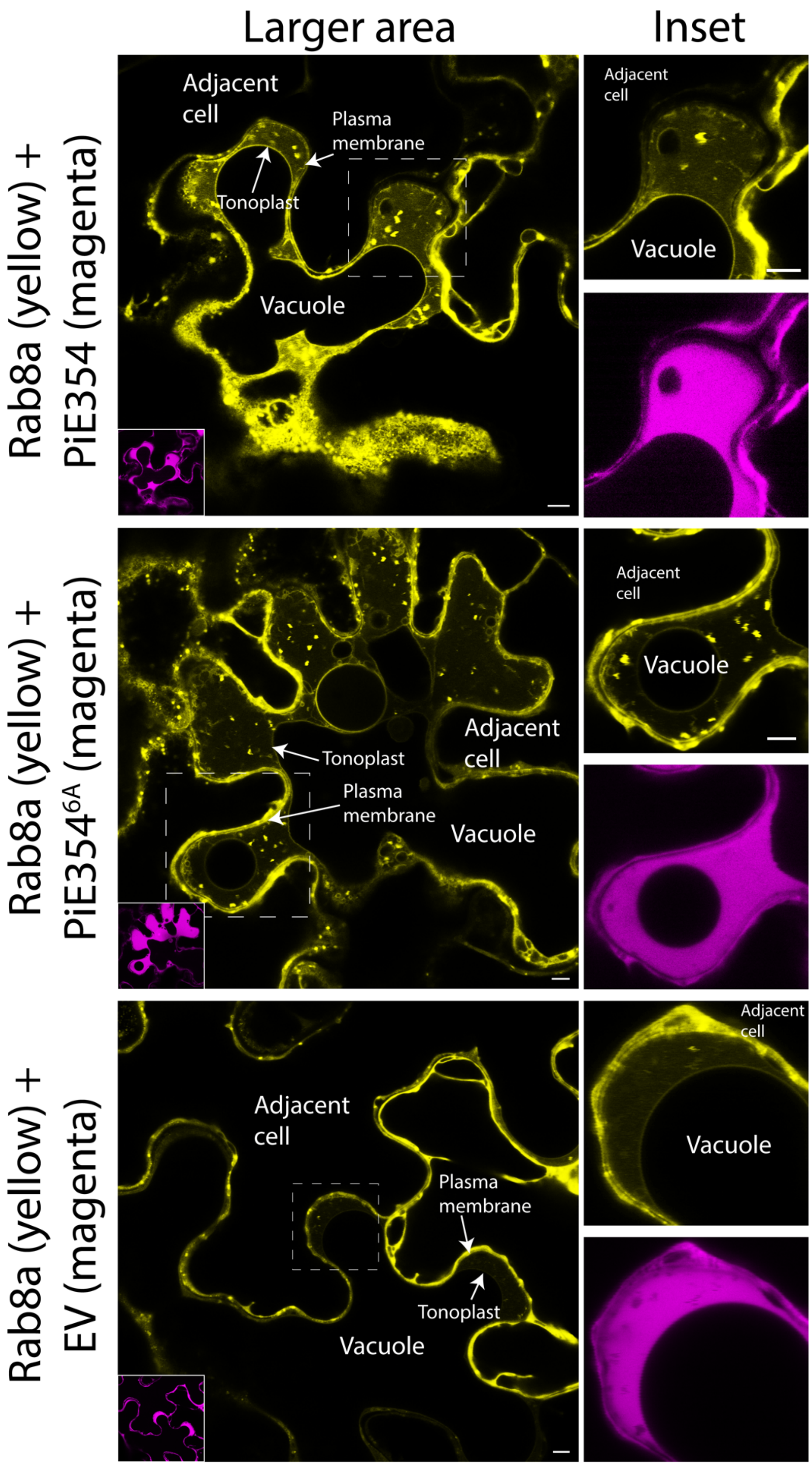

Larger area
Inset
Rab8a (yellow) + PiE354 (magenta)
Adjacent cell
Plasma membrane
Tonoplast
Vacuole
Rab8a (yellow) + PiE354^{6A} (magenta)
Adjacent cell
Tonoplast
Plasma membrane
Vacuole
Rab8a (yellow) + EV (magenta)
Adjacent cell
Plasma membrane
Tonoplast
Vacuole

Fig. S9. PiE354 diverts Rab8a localization from the plasma membrane to the tonoplast dependent on its interaction with TOPGAP. Additional representative images for Fig. 7D, showing a larger cell area. Confocal micrographs of *N. benthamiana* leaf epidermal cells transiently expressing either RFP:PiE354, RFP:PiE354^{6A}, or RFP:EV, with GFP:Rab8a. Presented images are single plane images. Scale bars, 5 μm. Insets are used to show a small portion of the larger image at higher magnification, as indicated by dashed white boxes. EV and PiE354^{6A} result in a predominant localization of Rab8a to the plasma membrane, while PiE354 re-directs the predominant localization of Rab8a to the tonoplast.

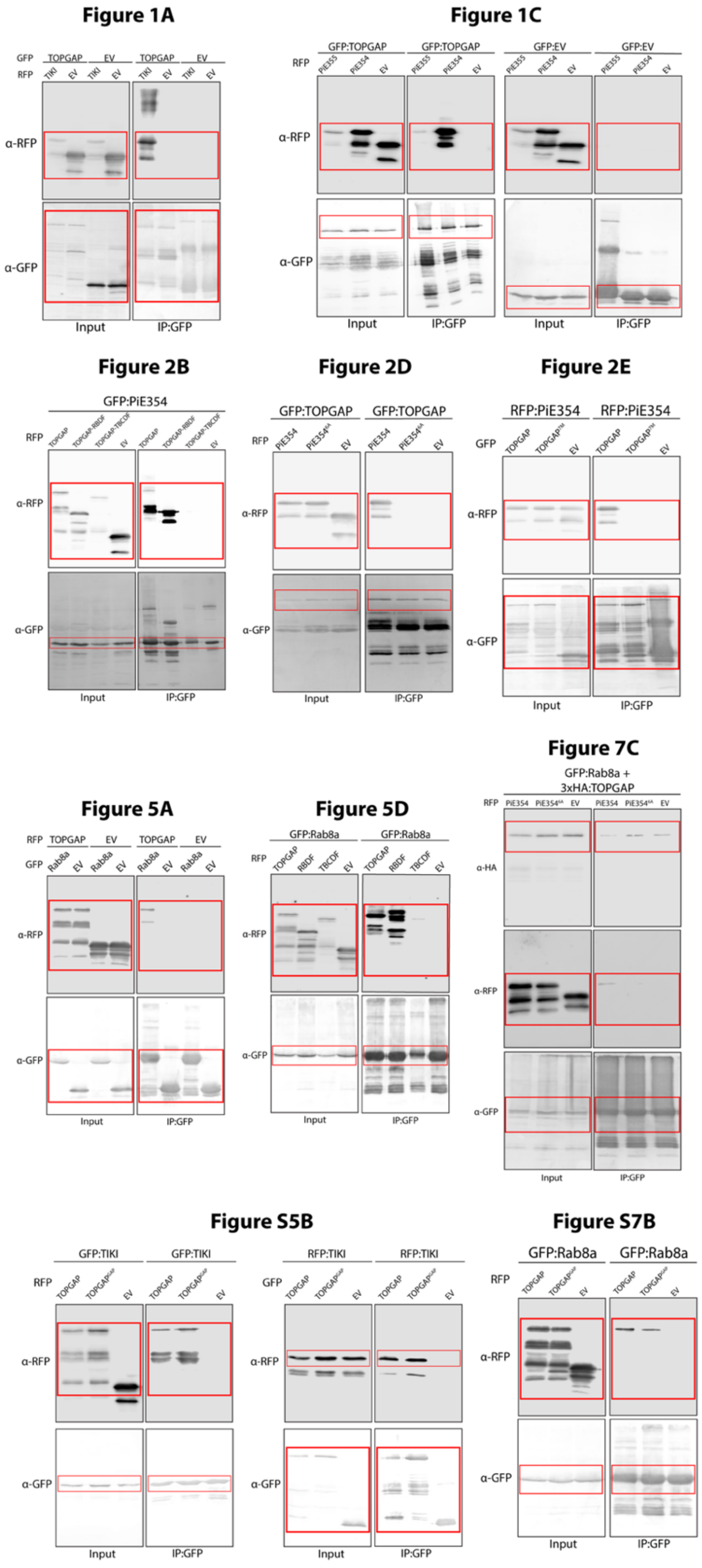

Fig. S10. Full-size western blots that were performed for this research.

Table S5. Primers and synthetic fragments used in this research.

Primer	Sequence
TOPGAP_F	CTGGATCTGGAGAATTTGATCACGAAATAGACCTCCACG
TOPGAP_R	TAGCATGGCCGCGGATTTACAATACATCATCATCGTCC
TOPGAP-GAPmut_R	CATCTTTTTCAATAAGACCCTTTCTTTCTC
TOPGAP-RBDF_R	TAGCATGGCCGCGGATTTACTCCAAGGAAGAGAGAGTTC
TOPGAP-TBCDF_F	CTGGATCTGGAGAATTTGATCTTCCGGGGGCTGTTTCTG
TOPGAP_RTPCR_F	GTCATTAGTGTGGGGAAAACC
TOPGAP_RTPCR_R	GTCACGCAAACACTTCACATTA
GAPDH_RTPCR_F	ATGGCTTCTCATGCAGCTTT
GAPDH_RTPCR_R	ATCCTGTGGTCTTGGGAGTG
**Synthetic Fragment**	**Sequence**
TOPGAP-GAPmut_synfrag	CAAAATTTCGAGAAAGAAAGGGTCTTATTGAAAAAGATGTGGTC<br>GCTACTGATAGGTCGATTCCATTCTATGAAGGGGATGATAATTCT<br>AATGTGAAGTGTTTGCGTGACATACTGCTCACTTACTCTTTCTAC<br>AACTTTGACCTGGGCTACTGCGCTGGTATGAGTGATTTTCTTTCA<br>CCAATATTGTGTGTGATGGAAGATGAACCAGAATCATTTTGGTG<br>CTTTGTGGCATTGATGGAGCGGCTTGGACCAAATTTTAACCGTG<br>ACCAGAACGGAGTGCATTCTCAGCTTTTTGCATTGTCAAAGTTGG<br>TGGAGTTATTGGACAACCCTTTGCATGATTACTTCAAACAGAAG<br>GACTGCTTGAATTATTTCTTTTGCTTTCGCTGGGTTCTTATACAAT<br>TCAAAAGGGAGTTTGACTTGGAGAAGACAATGCGGTTGTGGGAG<br>GTCTTATGGACACATTATTTGAGTGAGCATCTACATCTGTATGTC<br>TGTGTTGCAATTCTGAGAAGATATCGCAGTAAAATAATAGGAGA<br>AGAGATGGATTTCGACACGCTGCTGAAATTTATCAATGAGTTAA<br>GTGGTCATATTGACCTGGATGCTACCGTCAGGGAATCTGAAGCT<br>TTGTTTATTTGTGCCGGTGAAAATGGTGAAGCTTGCATTCCTCCT<br>GGAACTCCACCTTCATTTCCATTTGAGGGTACTTCAATGTATTAC<br>CAACAGGACGATGATGATGTATTGTAAATCCCGCGGCCATGCTA<br>GAGTCCGCAAAAATCACCAGT
TOPGAP-7M_synfrag	CTGGATCTGGAGAATTTGATCACGAAATAGACCTCCACGATCTT<br>TCCGACGACGCCGATTACGCTGCTTCTATGCATCAAGGTTCGATG<br>AGCATGACTATGAGCAGTAGCAGTAAACCGAGTTCATCCAGGGA<br>ACAAGTTGGTGTTGAAATAGTGTATTTGAAAGATAATGTGGCAA<br>TACACCCGACTCAACATGGATGGGAGAGGATCAGAGGTCGGCTG<br>AAGCTGATCAAGCAAGGAAGTTCTCTGTTAATGACTTGGATTCC<br>ATATAAAGGGCAAAGCTCAAGTGCAAGGCTGTCTGAAAAAGAT<br>AAGAGTGCCTATACAATAAGAGCAGTGCCTTTCTCGGATATTAG<br>GTCAATCGCTAGACACACTCCTACAGCGGGTTGGGCGTATGCTA<br>TTATAGTTTTGTTGTCAGGACTAGGATTTCCTCCACTTGCTTTTTA<br>CAATGGTGGTCTCAAGGAATTTCTTGCAACGATTAAGCAACATG<br>CCTTTCTTGTGAGGTCTGCTGAAGATGCAAATATATTTCTTGTCA

	ATGACTTCCAAGACCCGCTCCAGAGAACTCTCTCTTCCTTGGAGC<br>TTCCGGGGGCTGTTTCTGTTGCAAACAGTCCAATTTCATCCGTTG<br>CACCCAGTGAATCTTCACCCACTTGGACAAACAGAGAAGCCCCT<br>GATAAGAGCTCCACATATATTCGACAAAATGGCAGTCAAAGACA<br>AAAACATAATGATCCTCGACATTTTTCTATTCAAGTACTGGAGA<br>AATTTTCTCTTGTCACCAGATTTGCTCGTGAAACAAAATCTCAAC<br>TTCTTCGTGAAGCTCATGGTGATGGTTTCATTTCTAATGCAAGGA<br>GGAAGCATGAAAAAAAGCCAAATAATTATTCTTTTGTTGTTGAA<br>TCTAATGATGCTCATGAGCCGCTTGAAGATGTTCCTGTGCCAGCA<br>GATTCTTTGGTGAAACGTTCTAGCGAGAAACATAGTCACAATGA<br>AGAAGAGGCATTGCGTGATGAAACTTTTGAGTATGAAAAATTGT<br>CATTAGTGTGGGGAAAACCGCGCCAGCCTCCGTTGGGATCAAAA<br>GAGTGGTCCACCTTTTTGGACTCTGAAGGGAGGATCATAGACTC<br>GCAGGCACTAAGAAAGAGGATCTTTTATGGAGGAGTGGAGAAA<br>GGTCTGAGGAAAGAGGTCTGGAGATTTCTGTTGGGATATCACTC<br>GTATGATTCAACGTATGCTGAGAGGAAATACCTTGCATCTATCA<br>AAAAGTCAGAGTATGAAACATTAAAGAACCAGTGGAAGAGCAT<br>CTCTAAAGAGCAGGCCAAAAGATTTACAAAATTTCGAGAAAGA<br>AAGGGTCTTATTGAAAAAGATGTGGTCAGGACTGATAGGTCGAT<br>TCCATTCTATGAAGGGGATGATAATTCTAATGTGAAGTGTTTGCG<br>TGACATACTGCTCACTTACTCTTTCTACAACTTTGACCTGGGCTA<br>CTGCCAGGGTATGAGTGATTTTCTTTCACCAATATTGTGTGTGAT<br>GGAAGATGAACCAGAATCATTTTGGTGCTTTGTGGCATTGATGG<br>AGCGGCTTGGACCAAATTTTAACCGTGACCAGAACGGAGTGCAT<br>TCTCAGCTTTTTGCATTGTCAAAGTTGGTGGAGTTATTGGACAAC<br>CCTTTGCATGATTACTTCAAACAGAAGGACTGCTTGAATTATTTC<br>TTTTGCTTTCGCTGGGTTCTTATACAATTCAAAAGGGAGTTTGAC<br>TTGGAGAAGACAATGCGGTTGTGGGAGGTCTTATGGACACATTA<br>TTTGAGTGAGCATCTACATCTGTATGTCTGTGTTGCAATTCTGAG<br>AAGATATCGCAGTAAAATAATAGGAGAAGAGATGGATTTCGAC<br>ACGCTGCTGAAATTTATCAATGAGTTAAGTGGTCATATTGACCTG<br>GATGCTACCGTCAGGGAATCTGAAGCTTTGTTTATTTGTGCCGGT<br>GAAAATGGTGAAGCTTGCATTCCTCCTGGAACTCCACCTTCATTT<br>CCATTTGAGGGTACTTCAATGTATTACCAACAGGACGATGATGA<br>TGTATTGTAAATCCCGCGGCCATGCTA
TIKI_synfrag	ATGGACTACAAGGACGACGATGACAAATCGGATTCTCAGCACAA<br>TCAGCTCCTCCCGCACGCGATCAAGAGTGCTACCCAAGAGAAGA<br>TGAGATTCCTAAGAAGCCACAAAACAGAAGAGGAGAGGACCTT<br>CAATACCAACAACTACCCCTTCCTGATGACTGAAGCGGCGGCTA<br>ATGAAATGACGAAACGGGTAGATGAAGTGTTCTCTGGGAAGCTC<br>CAGGAAATTACCAGGGGTAAAGATATTCTGACGCGTTGGAAGAA<br>TGAGGGATTAACGCTTTCCCAAATTAAGTCTTTGTTGAAAGAAG<br>CCGGTAAGTGGCACAGTACAAGTGAGCGCGAGGTCTACAAACTG<br>CTTAAGAACGGTCCATAA
PiE354_synfrag	GGATCTGCTGGATCTGCTGCTGGATCTGGAGAATTTGATGTTCAT<br>CCTCTTCTTATGCATGAAGGTGTTGCTGTTGCTATTGCTAAGTCT

	GTTTCTGCTGATCTTGATTCTAGACTTATGGCTAAGGTTTCTCAA<br>ATTGATAATGGTAAGGAAATTCTTAAGCAATGGAGAGATAAGGG<br>TCTTACTATTGCTAAGCTTAAGCCTTTGCTTAAGAATACTAAGAA<br>GTGGAAGGCTACTCCTGAAAGAGCTGTTTATAATCTTCTCAAGA<br>AGGAAAGATTTTCTTAGATCCCGCGGCCATGCTAGAGTCCGCAA<br>AAATCACCAGT
PiE355_synfrag	GGATCTGCTGGATCTGCTGCTGGATCTGGAGAATTTGATGGTAG<br>ATATGATTTTCCTTTTCTTGTTTCTGAAGGTGCTGCTAGAGAAAT<br>GGCTAAGAGAATTTCTGATAATCTTGATTCTACTCTTATGTCTAG<br>ACTTTCTGCTATTTCTAATGGTAAGACTATTCTTAATAGATGGAG<br>AAATGAGGGCCTTTCTCTTGCTGAAGTTAAGCCTCTTCTTAAGGA<br>AGCTGGTAAGTGGGGTGATACTTCTGAAAGAGCTGTTTATAAGC<br>TTCTTAAGTCTGAGGGTCTTTCTTAGATCCCGCGGCCATGCTAGA<br>GTCCGCAAAAATCACCAGT
PiE354-6A_synfrag	GGATGCGCTGGATCTGCTGCTGGATCTGGAGAATTTGATGTTCAT<br>CCTGCTCTTGCGCATGCAGGTGTTGCTGTTGCTATTGCTAAGTCT<br>GTTTCTGCTGCTCTTGATTCTAGACTTATGGCTAAGGTTTCTCAA<br>ATTGATAATGGTAAGGAAATTCTTAAGCAATGGGCAGATAAGGG<br>TCTTACTATTGCTAAGCTTAAGCCTTTGCTTAAGAATACTAAGAA<br>GTGGAAGGCTACTCCTGAAAGAGCTGTTTATAATCTTCTCAAGA<br>AGGAAAGATTTGCTTAGATCCCGCGGCCATGCTAGAGTCCGCAA<br>AAATCACCGCT
TOPGAP-silencing_synfrag	ACCAGGTCTCAGGAGTTGCGTGATATACTGCTGACTTACTCATTC<br>TACAACTTTGATTTGGGTTACTGTCAGGGTATGAGTGATCTCCTT<br>TCACCAATATTATATGTCATGGAAGATGAATCAGAATCATTTTG<br>GTGCTTTGTGGCATTGATGGAACGACTGGGGCCGAACTTCAACC<br>GTGACCAAAATGGAATGCACTCTCAGCTTTTTGCATTATCAAAGT<br>TGGTGGAATTGGAGTTTGAATTTGAGAAAACAATGCGCAATTCT<br>GAGAAGATATCGCAGTAAAATAATAGGAGAAGAGATGGATTTC<br>GACACGCTGCTGAAATTTATCAATGAGTTAAGTGGTCATATTGA<br>CCTGGATGCTACCGTCAGGGAATCTGAAGCTTTGTTTATTTGTGC<br>CGGTGAAAATGGTGAAGCTTGCATTCCTCCTGGAACTCCACCTTC<br>ATTTCCATTTGAGGGTACTTCAATGTATTACCAACAGGACGATG<br>ATGATGTATTGTAAACGATGAGACCTGGT

Table S6. Detail of all constructs used in this research.

Construct	Vector backbone	Ref.	Primers/Synthetic Fragments used
GFP:TOPGAP	pK7WGF2 domesticated for Gibson	This work	Primers: TOPGAP_F and TOPGAP_R
RFP:TOPGAP	pK7WGF2 domesticated for Gibson	This work	Primers: TOPGAP_F and TOPGAP_R
3xHA:TOPGAP	pK7WGF2 domesticated for Gibson	This work	Primers: TOPGAP_F and TOPGAP_R
GFP:TOPGAP-7M	pK7WGF2 domesticated for Gibson	This work	Synthetic fragment: TOPGAP-7M_synfrag
GFP:TOPGAP-GAPmut	pK7WGF2 domesticated for Gibson	This work	Primers: TOPGAP_F and TOPGAP-GAPmut_R, and Synthetic fragment: TOPGAP-GAPmut_synfrag
RFP:TOPGAP-GAPmut	pK7WGF2 domesticated for Gibson	This work	Primers: TOPGAP_F and TOPGAP-GAPmut_R, and Synthetic fragment: TOPGAP-GAPmut_synfrag
RFP:TOPGAP-RBDF	pK7WGF2 domesticated for Gibson	This work	Primers: TOPGAP_F and TOPGAP-RBDF_R
RFP:TOPGAP-TBCDF	pK7WGF2 domesticated for Gibson	This work	Primers: TOPGAP-TBCDF_F and TOPGAP_R
RFP:FLAG:TIKI	pUC57	This work	Synthetic fragment: TIKI_synfrag
GFP:PiE354	pK7WGF2 domesticated for Gibson	This work	Synthetic fragment: PiE354_synfrag
RFP:PiE354	pK7WGF2 domesticated for Gibson	This work	Synthetic fragment: PiE354_synfrag
GFP:PiE354-6A	pK7WGF2 domesticated for Gibson	This work	Synthetic fragment: PiE354-6A_synfrag
RFP:PiE354-6A	pK7WGF2 domesticated for Gibson	This work	Synthetic fragment: PiE354-6A_synfrag
RFP:PiE355	pK7WGF2 domesticated for Gibson	This work	Synthetic fragment: PiE355_synfrag
GFP:EV	pK7WGF2 domesticated for Gibson	(*38*)	

RFP:EV	pK7WGF2 domesticated for Gibson	(*38*)	
GFP:Rab8a	pK7WGF2 domesticated for Gibson	(*20*)	
NRC4:GFP	pK7WGF2 domesticated for Gibson	(*54*)	
Rx:HA	pBI	(*55*)	
CP:csBP	pBIN61	(*55*)	
RNAi:TOPGAP	pRNAi-GG	This work	Synthetic fragment: TOPGAP-silencing_synfrag
RNAi:Rab8a	pRNAi-GG	(*20*)	
RNAi:GUS	pRNAi-GG	(*20*)	

Other Supplementary Materials for this manuscript include the following:

Table S1. Protein and DNA sequences of effectors.
Table S2. List of interactors of TIKI identified in Y2H.
Table S3. List of interactors of TIKI identified by IP-MS.
Table S4. List of interactors of TOPGAP identified by IP-MS.
Table S7. Proteins and sequences used for AF2.
Table S8. Antibodies used in this research.
Table S9. Statistics details and summary.

REFERENCES AND NOTES

1. T. O. Bozkurt, S. Schornack, J. Win, T. Shindo, M. Ilyas, R. Oliva, L. M. Cano, A. M. Jones, E. Huitema, R. A. van der Hoorn, S. Kamoun, *Phytophthora infestans* effector AVRblb2 prevents secretion of a plant immune protease at the haustorial interface. *Proc. Natl. Acad. Sci. U.S.A.* **108**, 20832–20837 (2011).

2. K. Nomura, C. Mecey, Y. N. Lee, L. A. Imboden, J. H. Chang, S. Y. He, Effector-triggered immunity blocks pathogen degradation of an immunity-associated vesicle traffic regulator in *Arabidopsis*. *Proc. Natl. Acad. Sci. U.S.A.* **108**, 10774–10779 (2011).

3. T. Li, G. Ai, X. Fu, J. Liu, H. Zhu, Y. Zhai, W. Pan, D. Shen, M. Jing, A. Xia, D. Dou, A *Phytophthora capsici* RXLR effector manipulates plant immunity by targeting RAB proteins and disturbing the protein trafficking pathway. *Mol. Plant Pathol.* **23**, 1721–1736 (2022).

4. I. Tomczynska, M. Stumpe, F. Mauch, A conserved RxLR effector interacts with host RABA-type GTPases to inhibit vesicle-mediated secretion of antimicrobial proteins. *Plant J.* **95**, 187–203 (2018).

5. V. A. Michalopoulou, G. Mermigka, K. Kotsaridis, A. Mentzelopoulou, P. H. N. Celie, P. N. Moschou, J. D. G. Jones, P. F. Sarris, The host exocyst complex is targeted by a conserved bacterial type-III effector that promotes virulence. *Plant Cell* **34**, 3400–3424 (2022).

6. B. Petre, M. P. Contreras, T. O. Bozkurt, M. H. Schattat, J. Sklenar, S. Schornack, A. Abd-El-Haliem, R. Castells-Graells, R. Lozano-Duran, Y. F. Dagdas, F. L. H. Menke, A. M. E. Jones, J. H. Vossen, S. Robatzek, S. Kamoun, J. Win, Host-interactor screens of *Phytophthora infestans* RXLR proteins reveal vesicle trafficking as a major effector-targeted process. *Plant Cell* **33**, 1447–1471 (2021).

7. Y. F. Dagdas, P. Pandey, Y. Tumtas, N. Sanguankiattichai, K. Belhaj, C. Duggan, A. Y. Leary, M. E. Segretin, M. P. Contreras, Z. Savage, V. S. Khandare, S. Kamoun, T. O. Bozkurt, Host autophagy machinery is diverted to the pathogen interface to mediate focal defense responses against the Irish potato famine pathogen. *eLife* **7**, e37476 (2018).

8. C. Kwon, C. Neu, S. Pajonk, H. S. Yun, U. Lipka, M. Humphry, S. Bau, M. Straus, M. Kwaaitaal, H. Rampelt, F. El Kasmi, G. Jurgens, J. Parker, R. Panstruga, V. Lipka, P. Schulze-Lefert, Co-option of a default secretory pathway for plant immune responses. *Nature* **451**, 835–840 (2008).

9. M. E. Nielsen, H. Thordal-Christensen, Transcytosis shuts the door for an unwanted guest. *Trends Plant Sci.* **18**, 611–616 (2013).

10. E. L. H. Yuen, S. Shepherd, T. O. Bozkurt, Traffic control: Subversion of plant membrane trafficking by pathogens. *Annu. Rev. Phytopathol.* **61**, 325–350 (2023).

11. S. Shepherd, E. L. H. Yuen, P. Carella, T. O. Bozkurt, The wheels of destruction: Plant NLR immune receptors are mobile and structurally dynamic disease resistance proteins. *Curr. Opin. Plant Biol.* **74**, 102372 (2023).

12. Z. Savage, C. Duggan, A. Toufexi, P. Pandey, Y. Liang, M. E. Segretin, L. H. Yuen, D. C. A. Gaboriau, A. Y. Leary, Y. Tumtas, V. Khandare, A. D. Ward, S. W. Botchway, B. C. Bateman, I. Pan, M. Schattat, I. Sparkes, T. O. Bozkurt, Chloroplasts alter their morphology and accumulate at the pathogen interface during infection by *Phytophthora infestans*. *Plant J.* **107**, 1771–1787 (2021).

13. E. L. H. Yuen, Z. Savage, V. Adamkova, C. Vuolo, Y. Zhou, Y. Tumtas, J. L. Erickson, J. Prautsch, A. I. Balmez, J. Stuttmann, C. Duggan, F. Rivetti, C. Molinari, M. Schattat,T. O. Bozkurt, Membrane contact sites between chloroplasts and pathogen interface underpin plant focal immune responses. bioRxiv 463641 [Preprint] (2024). https://doi. org/10.1101/2021.10.08.463641.

14. T. O. Bozkurt, K. Belhaj, Y. F. Dagdas, A. Chaparro-Garcia, C. H. Wu, L. M. Cano, S. Kamoun, Rerouting of plant late endocytic trafficking toward a pathogen interface. *Traffic* **16**, 204–226 (2015).

15. T. O. Bozkurt, S. Kamoun, The plant-pathogen haustorial interface at a glance. *J. Cell Sci.* **133**, jcs237958 (2020).

16. F. J. King, E. L. H. Yuen, T. O. Bozkurt, Border control: Manipulation of the host-pathogen interface by perihaustorial oomycete effectors. *Mol. Plant Microbe Interact.* **37**, 220–226 (2024).

17. N. Inada, S. Betsuyaku, T. L. Shimada, K. Ebine, E. Ito, N. Kutsuna, S. Hasezawa, Y. Takano, H. Fukuda, A. Nakano, T. Ueda, Modulation of plant RAB GTPase-mediated membrane trafficking pathway at the interface between plants and obligate biotrophic pathogens. *Plant Cell Physiol.* **57**, 1854–1864 (2016).

18. K. Ebine, M. Fujimoto, Y. Okatani, T. Nishiyama, T. Goh, E. Ito, T. Dainobu, A. Nishitani, T. Uemura, M. H. Sato, H. Thordal-Christensen, N. Tsutsumi, A. Nakano, T. Ueda, A membrane trafficking pathway regulated by the plant-specific RAB GTPase ARA6. *Nat. Cell Biol.* **13**, 853–859 (2011).

19. E. Nielsen, A. Y. Cheung, T. Ueda, The regulatory RAB and ARF GTPases for vesicular trafficking. *Plant Physiol.* **147**, 1516–1526 (2008).

20. P. Pandey, A. Y. Leary, Y. Tumtas, Z. Savage, B. Dagvadorj, C. Duggan, E. L. Yuen, N. Sanguankiattichai, E. Tan, V. Khandare, A. J. Connerton, T. Yunusov, M. Madalinski, F. G. Mirkin, S. Schornack, Y. Dagdas, S. Kamoun, T. O. Bozkurt, An oomycete effector subverts host vesicle trafficking to channel starvation-induced autophagy to the pathogen interface. *eLife* **10**, e65285 (2021).

21. R. M. Nottingham, S. R. Pfeffer, Defining the boundaries: Rab GEFs and GAPs. *Proc. Natl. Acad. Sci. U.S.A.* **106**, 14185–14186 (2009).

22. C. Gabernet-Castello, A. J. O'Reilly, J. B. Dacks, M. C. Field, Evolution of Tre-2/Bub2/Cdc16 (TBC) Rab GTPase-activating proteins. *Mol. Biol. Cell* **24**, 1574–1583 (2013).

23. L. De Arras, I. V. Yang, B. Lackford, D. W. Riches, R. Prekeris, J. H. Freedman, D. A. Schwartz, S. Alper, Spatiotemporal inhibition of innate immunity signaling by the Tbc1d23 RAB-GAP. *J. Immunol.* **188**, 2905–2913 (2012).

24. J. Roos, S. Bejai, S. Oide, C. Dixelius, *RabGAP22* is required for defense to the vascular pathogen *Verticillium longisporum* and contributes to stomata immunity. *PLOS ONE* **9**, e88187 (2014).

25. K. Nomura, S. Debroy, Y. H. Lee, N. Pumplin, J. Jones, S. Y. He, A bacterial virulence protein suppresses host innate immunity to cause plant disease. *Science* **313**, 220–223 (2006).

26. S. Ustun, A. Hafren, Q. Liu, R. S. Marshall, E. A. Minina, P. V. Bozhkov, R. D. Vierstra, D. Hofius, Bacteria exploit autophagy for proteasome degradation and enhanced virulence in plants. *Plant Cell* **30**, 668–685 (2018).

27. A. K. Machado Wood, V. Panwar, M. Grimwade-Mann, T. Ashfield, K. E. Hammond-Kosack, K. Kanyuka, The vesicular trafficking system component MIN7 is required for minimizing *Fusarium graminearum* infection. *J. Exp. Bot.* **72**, 5010–5023 (2021).

28. H. McLellan, S. E. Harvey, J. Steinbrenner, M. R. Armstrong, Q. He, R. Clewes, L. Pritchard, W. Wang, S. Wang, T. Nussbaumer, B. Dohai, Q. Luo, P. Kumari, H. Duan, A. Roberts, P. C. Boevink, C. Neumann, N. Champouret, I. Hein, P. Falter-Braun, J. Beynon, K. Denby, P. R. J. Birch, Exploiting breakdown in nonhost effector-target interactions to boost host disease resistance. *Proc. Natl. Acad. Sci. U.S.A.* **119**, e2114064119 (2022).

29. L. S. Boutemy, S. R. F. King, J. Win, R. K. Hughes, T. A. Clarke, T. M. A. Blumenschein, S. Kamoun, M. J. Banfield, Structures of *Phytophthora* RXLR effector proteins: A conserved but adaptable fold underpins functional diversity. *J. Biol. Chem.* **286**, 35834–35842 (2011).

30. Z. Zhang, S. Wang, T. Shen, J. Chen, J. Ding, Crystal structure of the Rab9A-RUTBC2 RBD complex reveals the molecular basis for the binding specificity of Rab9A with RUTBC2. *Structure* **22**, 1408–1420 (2014).

31. X. Pan, S. Eathiraj, M. Munson, D. G. Lambright, TBC-domain GAPs for Rab GTPases accelerate GTP hydrolysis by a dual-finger mechanism. *Nature* **442**, 303–306 (2006).

32. K. I. Kurotani, H. Hirakawa, K. Shirasawa, Y. Tanizawa, Y. Nakamura, S. Isobe, M. Notaguchi, Genome sequence and analysis of *Nicotiana benthamiana*, the model plant for interactions between organisms. *Plant Cell Physiol.* **64**, 248–257 (2023).

33. E. L. H. Yuen, A. Y. Leary, M. Clavel, Y. Tumtas, A. Mohseni, J. Zhao, L. Picchianti, M. Jamshidiha, P. Pandey, C. Duggan, E. Cota, Y. Dagdas, T. O. Bozkurt, A RabGAP negatively regulates plant autophagy and immune trafficking. *Curr. Biol.* **34**, 2049–2065e46 (2024).

34. L. Henry, D. R. Sheff, Rab8 regulates basolateral secretory, but not recycling, traffic at the recycling endosome. *Mol. Biol. Cell* **19**, 2059–2068 (2008).

35. E. B. Speth, L. Imboden, P. Hauck, S. Y. He, Subcellular localization and functional analysis of the Arabidopsis GTPase RabE. *Plant Physiol.* **149**, 1824–1837 (2009).

36. J. Win, A. Chaparro-Garcia, K. Belhaj, D. G. Saunders, K. Yoshida, S. Dong, S. Schornack, C. Zipfel, S. Robatzek, S. A. Hogenhout, S. Kamoun, Effector biology of plant-associated organisms: Concepts and perspectives. *Cold Spring Harb. Symp. Quant. Biol.* **77**, 235–247 (2012).

37. Y. F. Dagdas, K. Belhaj, A. Maqbool, A. Chaparro-Garcia, P. Pandey, B. Petre, N. Tabassum, N. Cruz-Mireles, R. K. Hughes, J. Sklenar, J. Win, F. Menke, K. Findlay, M. J. Banfield, S. Kamoun, T. O. Bozkurt, An effector of the Irish potato famine pathogen antagonizes a host autophagy cargo receptor. *eLife* **5**, e10856 (2016).

38. P. Yan, W. Shen, X. Gao, X. Li, P. Zhou, J. Duan, High-throughput construction of intron-containing hairpin RNA vectors for RNAi in plants. *PLOS ONE* **7**, e38186 (2012).

39. P. van West, A. J. de Jong, H. S. Judelson, A. M. Emons, F. Govers, The *ipiO* gene of *Phytophthora infestans* is highly expressed in invading hyphae during infection. *Fungal Genet. Biol.* **23**, 126–138 (1998).

40. M. Mirdita, K. Schutze, Y. Moriwaki, L. Heo, S. Ovchinnikov, M. Steinegger, ColabFold: Making protein folding accessible to all. *Nat. Methods* **19**, 679–682 (2022).

41. J. Jumper, R. Evans, A. Pritzel, T. Green, M. Figurnov, O. Ronneberger, K. Tunyasuvunakool, R. Bates, A. Zidek, A. Potapenko, A. Bridgland, C. Meyer, S. A. A. Kohl, A. J. Ballard, A. Cowie, B. Romera-Paredes, S. Nikolov, R. Jain, J. Adler, T. Back, S. Petersen, D. Reiman, E. Clancy, M. Zielinski, M. Steinegger, M. Pacholska, T. Berghammer, S. Bodenstein, D. Silver, O. Vinyals, A. W. Senior, K. Kavukcuoglu, P. Kohli, D. Hassabis, Highly accurate protein structure prediction with AlphaFold. *Nature* **596**, 583–589 (2021).

42. V. Mariani, M. Biasini, A. Barbato, T. Schwede, lDDT: A local superposition-free score for comparing protein structures and models using distance difference tests. *Bioinformatics* **29**, 2722–2728 (2013).

43. R. C. Edgar, MUSCLE: Multiple sequence alignment with high accuracy and high throughput. *Nucleic Acids Res.* **32**, 1792–1797 (2004).

44. X. Robert, P. Gouet, Deciphering key features in protein structures with the new ENDscript server. *Nucleic Acids Res.* **42**, W320–W324 (2014).

45. C. Camacho, G. Coulouris, V. Avagyan, N. Ma, J. Papadopoulos, K. Bealer, T. L. Madden, BLAST+: Architecture and applications. *BMC Bioinformatics* **10**, 421 (2009).

46. F. Sievers, A. Wilm, D. Dineen, T. J. Gibson, K. Karplus, W. Li, R. Lopez, H. McWilliam, M. Remmert, J. Soding, J. D. Thompson, D. G. Higgins, Fast, scalable generation of high-quality protein multiple sequence alignments using Clustal Omega. *Mol. Syst. Biol.* **7**, 539 (2011).

47. K. P. Schliep, Phangorn: Phylogenetic analysis in R. *Bioinformatics* **27**, 592–593 (2011).

48. G. Yu, D. K. Smith, H. Zhu, Y. Guan, T. T.-Y. Lam, ggtree: An r package for visualization and annotation of phylogenetic trees with their covariates and other associated data. *Methods Ecol. Evol.* **8**, 28–36 (2017).

49. P. Stothard, The sequence manipulation suite: JavaScript programs for analyzing and formatting protein and DNA sequences. *Biotechniques* **28**, 1102–1104 (2000).

50. B. M. O'Leary, A. Rico, S. McCraw, H. N. Fones, G. M. Preston, The infiltration-centrifugation technique for extraction of apoplastic fluid from plant leaves using *Phaseolus vulgaris* as an example. *J. Vis. Exp.* **19**, 52113 (2014).

51. C. H. Wu, A. Abd-El-Haliem, T. O. Bozkurt, K. Belhaj, R. Terauchi, J. H. Vossen, S. Kamoun, NLR network mediates immunity to diverse plant pathogens. *Proc. Natl. Acad. Sci. U.S.A.* **114**, 8113–8118 (2017).

52. H. Wickham, *ggplot2: Elegant Graphics for Data Analysis* (Springer International Publishing, 2016).

53. S. Wawra, F. Trusch, A. Matena, K. Apostolakis, U. Linne, I. Zhukov, J. Stanek, W. Kozminski, I. Davidson, C. J. Secombes, P. Bayer, P. van West, The RxLR motif of the host targeting effector AVR3a of *Phytophthora infestans* is cleaved before secretion. *Plant Cell* **29**, 1184–1195 (2017).

54. C. Duggan, E. Moratto, Z. Savage, E. Hamilton, H. Adachi, C. H. Wu, A. Y. Leary, Y. Tumtas, S. M. Rothery, A. Maqbool, S. Nohut, T. R. Martin, S. Kamoun, T. O. Bozkurt, Dynamic localization of a helper NLR at the plant-pathogen interface underpins pathogen recognition. *Proc Natl Acad Sci USA* **118**, e2104997118 (2021).

55. M. P. Contreras, H. Pai, M. Selvaraj, A. Toghani, D. M. Lawson, Y. Tumtas, C. Duggan, E. L. H. Yuen, C. E. M. Stevenson, A. Harant, A. Maqbool, C. H. Wu, T. O. Bozkurt, S. Kamoun, L. Derevnina, Resurrection of plant disease resistance proteins via helper NLR bioengineering.*Sci Adv* **9**, eadg3861 (2023).
